# Phase Transitions in Poly(vinylidene fluoride)/Polymethylene-Based Diblock Copolymers and Blends

**DOI:** 10.3390/polym13152442

**Published:** 2021-07-24

**Authors:** Nicolás María, Jon Maiz, Daniel E. Martínez-Tong, Angel Alegria, Fatimah Algarni, George Zapzas, Nikos Hadjichristidis, Alejandro J. Müller

**Affiliations:** 1 POLYMAT, University of the Basque Country UPV/EHU, Avenida de Tolosa 72, 20018 Donostia-San Sebastián, Spain; nicolas.maria@polymat.eu; 2 Centro de Física de Materiales (CFM) (CSIC-UPV/EHU)-Matrials Physics Center (MPC), Paseo Manuel de Lardizabal 5, 20018 Donostia-San Sebastián, Spain; danielenrique.martinezt@ehu.eus (D.E.M.-T.); angel.alegria@ehu.eus (A.A.); 3 IKERBASQUE—Basque Foundation for Science, Plaza Euskadi 5, 48009 Bilbao, Spain; 4 Department of Polymers and Advanced Materials: Physics, Chemistry and Technology, University of the Basque Country UPV/EHU, Paseo Manuel de Lardizabal 3, 20018 Donostia-San Sebastián, Spain; 5 KAUST Catalysis Center, Polymer Synthesis Laboratory, Physical Sciences and Engineering Division, King Abdullah University of Science and Technology (KAUST), Thuwal 23955-6900, Saudi Arabia; fatimah.algarni@kaust.edu.sa (F.A.); georgios.zapsas@kaust.edu.sa (G.Z.)

**Keywords:** poly(vinylidene fluoride)/polymethylene, blends, diblock copolymers, ferroelectric phase

## Abstract

The crystallization and morphology of two linear diblock copolymers based on polymethylene (PM) and poly(vinylidene fluoride) (PVDF) with compositions PM_23_-b-PVDF_77_ and PM_38_-b-PVDF_62_ (where the subscripts indicate the relative compositions in wt%) were compared with blends of neat components with identical compositions. The samples were studied by SAXS (Small Angle X-ray Scattering), WAXS (Wide Angle X-ray Scattering), PLOM (Polarized Light Optical Microscopy), TEM (Transmission Electron Microscopy), DSC (Differential Scanning Calorimetry), BDS (broadband dielectric spectroscopy), and FTIR (Fourier Transform Infrared Spectroscopy). The results showed that the blends are immiscible, while the diblock copolymers are miscible in the melt state (or very weakly segregated). The PVDF component crystallization was studied in detail. It was found that the polymorphic structure of PVDF was a strong function of its environment. The number of polymorphs and their amount depended on whether it was on its own as a homopolymer, as a block component in the diblock copolymers or as an immiscible phase in the blends. The cooling rate in non-isothermal crystallization or the crystallization temperature in isothermal tests also induced different polymorphic compositions in the PVDF crystals. As a result, we were able to produce samples with exclusive ferroelectric phases at specific preparation conditions, while others with mixtures of paraelectric and ferroelectric phases.

## 1. Introduction

Nowadays, polymers are important materials that may be used to enhance the safety and the quality of the environment and reduce the human impact. For example, they may take relevance in the field of renewable energies or self-powered applications where new polymeric materials can substitute inorganic devices having the same or better yield at a lower cost and with less environmental impact [1,2,3]. Therefore, the current development in new technologies requires the research of new materials to achieve a balance between evolution and pollution. 

During the last years, poly(vinylidene fluoride) (PVDF) [4,5] and its copolymers [6,7,8] have been the most used polymers in electronic devices or renewable energies. PVDF has good mechanical properties, such as flexibility and low cost. Its biocompatibility with other polymers and/or an extremely high chemical resistance make this polymer a great option for this kind of applications [9]. The most important characteristics of PVDF, apart from the properties commented above, are its ferroelectricity, piezoelectricity, and pyroelectricity, resulting from the polarization of its C-F bonds [10]. Therefore, the most used applications for this kind of fluoropolymers are data storage devices [11,12], sensors [13] and/or energy harvesting devices [14]. 

Another relevant characteristic of PVDF is its polymorphism: PVDF can crystallize in at least four different phases (α, β, γ, and δ), and not all of these phases have the same polar or non-polar properties [15,16]. When PVDF crystallizes from the melt, the most common and stable phase is the α-phase. This phase has a trans-gauche conformation, Tg^+^Tg^−^Tg^+^Tg^−^, and it is paraelectric. The drawback of this non-polar phase is that the PVDF crystallizing in this crystalline form is not very useful for the applications mentioned above [17]. 0 In contrast, the β-phase, with a conformation in all the carbons are in trans configuration, TTTT, has the highest dipole moment and is a piezoelectric and ferroelectric material [18,19]. 1 Unfortunately, this phase is not the most stable one, and it is difficult to obtain. A lot of methods and efforts have been developed during these last years to crystallize this ferroelectric phase, from mechanical stress to PVDF-based mixtures or blends (either with other polymers and/or fillers) and the synthesis of different copolymers [20,21,22]. The γ-phase has a higher melting temperature than the two phases mentioned above, and it is also ferroelectric, but it has less polarity, and its chain conformation is three trans and one gauche conformation TTTg^+^TTTg^−^ [23]. Finally, the δ-phase has the same chain conformation than the α-phase. The only difference is that the δ-phase has each second chain rotated 180° around the chain axis, and this small change provides the ferroelectric property to this phase compared to the paraelectric α-phase [24,25].

There are several papers in the literature in which ferroelectric and piezoelectric properties were obtained in PVDF and in PVDF-based materials. One of the most employed methods to obtain the β-phase in PVDF films is stretching [20,26], where mechanical stress is applied to transform polymer crystals from an α-phase to a β-phase. In this process, the stretching temperature is one of the important parameters to be considered [27], but the conversion from α- to β-phase obtained by this method is not complete, and both phases coexist simultaneously in the PVDF films [28]. The preparation of PVDF-based blends is another method to achieve the polar β-phase in PVDF [21,29] directly. PVDF blended with poly(methyl methacrylate) (PMMA), for example, crystallizes directly in the β-phase when the crystallization process occurs from the melt [30,31]. The addition of different fillers PVDF is another alternative, for example, samples of PVDF-TrFE (polyvinylidene-trifluoroethylene) with modified ZnO particles can promote the crystallization of the β-phase in the copolymer [32], and when PVDF is mixed with less than 0.2% of multi-walled carbon nanotubes, it can crystallize in an almost pure β-phase [33]. 

Other alternatives are to produce PVDF-based graft or block copolymers [34]. Graft copolymers based on PVDF were studied in order to improve the crystallization of the β-phase. Synthesis of PVDF grafted with poly (butylene succinate-co-adipate) (PVDF-*g*-PBSA) or poly (methyl methacrylate-co-acrylic acid) [PVDF-*g*-(PMMA-co-AA)] with previous ozonation of the PVDF induces the crystallization of the β-phase in almost 100%, thanks to the covalent links formed in the PVDF-OH groups [35]. Moreover, also block copolymers with PVDF were investigated to induce the β-phase. Beuermann et al. demonstrated by Fourier Transform Infrared Spectroscopy (FTIR) and Wide-Angle X-Ray Scattering (WAXS) that the PVDF crystallizes in the ferroelectric phase when PVDF-*b*-PMMA and PVDF-*b*-PS (Polystyrene) are synthesized [36,37]. In addition, in a previous work published by us, we have demonstrated that PVDF-*b*-PEO (Polyethylene oxide) block copolymers can crystallize only in the β-phase when the crystallization happens from the melt at low cooling rates, for instance, 1 °C/min [38].

In general, the properties of the blends and/or copolymers are different depending on the synthesis and the form in which they are present in the sample [39,40,41]. If the polymers are not compatible, the segregation observed in the material is different for blends and for copolymers. Segregation in blends happens on a larger scale due to the macro-phase segregation behavior [42]. Immiscible block copolymers cannot segregate into macro-phases due to their covalent bonds, but micro-phase segregation into regular domain patterns can occur [43]. Daoulas et al. have demonstrated by mesoscopic simulations that the differences between the block copolymers and blends in poly (*p*-phenylene vinylene) (PPV) and polyacrylate systems are due to this segregation phenomenon that makes the materials different for light-emitting diodes, so the final applications of both materials are not the same [44].

In this work, we study the crystallization of a polymethylene (PM) and PVDF system, polymers that are not miscible. We compare the PVDF homopolymer with two PM/PVDF blends and two PM-*b*-PVDF block copolymers in the same proportion in order to see the relevance of the segregation in the final properties of both materials. Using Differential Scanning Calorimetry (DSC), we study the behavior of these samples during the non-isothermal crystallization and during an isothermal process. Microscopy techniques and Small-Angle X-Ray Scattering (SAXS) are employed to study the miscibility between both polymers. Finally, the samples are fully characterized by Broadband Dielectric Spectroscopy (BDS), Fourier Transform Infrared Spectroscopy (FTIR), and Wide-Angle X-Ray Scattering (WAXS).

## 2. Materials and Methods

### 2.1. Materials

The diblock copolymers of polymethylene (PM) and poly(vinylidene fluoride) (PVDF) have been synthetized by Hadjichristidis et al. and published in a previous work [45]. In brief, the synthesis involves the following steps: (a) polyhomologation of dimethylsulfoxonium methylide using triethylborane as initiator followed by oxidation/hydrolysis to afford PM-OH,(b) esterification of the OH group with 2,2-bromoisobutyrylbromide to introduce bromide at the chain end, (c) halide exchange (Br→I) using sodium iodine to produce the macro-chain transfer agent (macro-CTA), and (d) Iodine transfer polymerization (ITP) of VDF with the macro-CTA and 1,1-bis(tert-butylperoxy)cyclohexa as the initiator (Scheme S1). The synthesis of polyvinylidene fluoride (PVDF) homopolymer has been accomplished via reversible addition−fragmentation chain-transfer polymerization (RAFT) polymerization by using (S-benzyl O-ethylxathate) as CTA and 1,1-bis(tert-butylperoxy)cyclohexane (Luperox 331P80, Sigma-Aldrich, Munich, Germany) as initiator. The synthesis and the characterization of the linear PVDF used in this study are given in the SI.

Blends were prepared by mixing the block copolymers with linear homopolymers, PM-OH and PVDF. The blends were prepared in the same compositions used for the block copolymers so that they could be compared. First, the PVDF and the PM mixtures were stirred until the total dissolution in cyclohexane during 24 h at 50 °C. Then, each mixture was drop-casted onto Teflon holders. Afterward, a fume hood was used to slowly evaporate the solvent, and finally, under vacuum conditions, the samples were well-dried in an oven at 40 °C for 72 h. All the polymers used in this work and their molecular characteristics are listed in Table 1.

### 2.2. Methods

#### 2.2.1. Differential Scanning Calorimetry (DSC)

A Perkin Elmer DSC 8000 equipment was used to carry out the DSC experiments. This equipment uses an Intracooler II as a cooling system. Before the measurements were performed, the equipment was calibrated using indium and tin standards. 

For the non-isothermal procedure, first, the samples were heated up to 20 °C above the highest melting temperature and held there for 3 min to ensure that the thermal history of the materials was completely erased. Then, samples were cooled at different cooling rates (60, 20, 5, and 1 °C/min) from the melt to 25 °C and then heated again to the molten state at a constant rate of 20 °C/min.

The protocol used to carry out the isothermal crystallization procedure was the same followed by Lorenzo et al. [46]. First, the minimum crystallization temperature (*T_c,_*_min_) was searched. To find it, samples were heated up to 20 °C above the melting temperature and held there for 3 min. Then, samples were cooled fast (at 60 °C/min) to a previously selected *T_c_*. When this *T_c_* was reached, samples were heated at 20 °C/min to the same melting temperature chosen in the previous step. When no peaks were observed in the subsequent heating scan, the *T_c_* mentioned in the second step was considered to be the minimum isothermal crystallization temperature [46].

The isothermal crystallization procedure consisted in a series of different steps. First, samples were melted at 20 °C above the melting temperature and held there for 3 min to erase the thermal history of the material. Then, samples were cooled down at 60 °C/min to the selected isothermal crystallization temperature and held at this *T_c_* for 40 min to achieve crystallization saturation. Once this crystallization process was finished, samples were heated at 20 °C/min to the previously selected melting temperature, and the process was reinitiated to the next programmed *T_c_* [46].

#### 2.2.2. X-ray Diffraction

Block copolymer samples were analyzed using Wide-Angle X-Ray Scattering (WAXS) and Small-Angle X-Ray Scattering (SAXS). These experiments were carried out in the ALBA Synchrotron facility using synchrotron radiation at the BL11-NCD beamline. Samples were measured in capillaries using a Linkam hot-stage system equipped with liquid nitrogen to control the temperature. The samples were melted at 200 °C for 3 min, then cooled down at the chosen cooling rate. The energy of the X-ray source was 12.4 keV (λ = 1.0 Å). The WAXS system configuration employed was a Rayonix LX255-HS sample detector with an active area of 230.4 mm × 76.8 mm. A sample to detector distance of 15.5 mm with a tilt angle of 27.3° was employed. The resulting pixel size was 44 μm^2^. For the SAXS experiments, the configuration was a Pilatus 1M sample detector, which had the following characteristics: active image area = 168.7 mm × 179.4 mm, the total number of pixels = 981 × 1043, pixel size = 172 µm × 172 µm, rate = 25 frames/sec and the distance used was 6463 mm.

#### 2.2.3. Polarized Light Optical Microscopy (PLOM)

All samples were analyzed by an Olympus BX51 polarized optical microscope coupled to a Linkam hot-stage that uses nitrogen to control the temperature and manages the cooling rate. An Olympus SC50 camera linked to the microscope was employed to observe the samples and take micrographs. Samples were dissolved in acetone or cyclohexane, and drops of the solutions were placed on a glass substrate and dried at room temperature.

#### 2.2.4. Fourier Transform Infrared Spectroscopy (FTIR)

A Nicolet 6700 Fourier Transform Infrared Spectrometer equipped with an Attenuated Total Reflectance (ATR) Golden Gate MK II with a diamond crystal was employed to analyze the samples. Samples were melted directly from the bulk at 200 °C in a Linkam hot-stage and then cooled down at 1 °C/min employing N_2_ in the cooling process. FTIR measurements were carried out after the cooling process at room temperature.

#### 2.2.5. Transmission Electron Microscopy (TEM)

All samples were stained with RuO_4_ before the measurements by immersing thin strips of material in this solution for 16 h. Then, the samples were cut in ultra-thin sections at room temperature with a diamond knife on a Leica EMFC6 ultra-microtome device. These 90 nm thick ultra-thin sections were mounted on a 200 mesh copper grid and then observed by a TECNAI G2 20 TWIN TEM equipped with a LaB6 filament operating at an accelerating voltage of 120 kV.

#### 2.2.6. Broadband Dielectric Spectroscopy (BDS)

The complex dielectric permittivity, ε* (ω) = ε′ (ω) − iε″ (ω), where ε′ is the real part and ε″ is the imaginary part, was obtained as a function of the frequency (ω) and temperature (*T*) by using a Novocontrol high-resolution dielectric analyzer (Alpha analyzer) (Novocontrol, Montabaur, Germany). The sample cell was set in a cryostat, whose temperature was controlled via a nitrogen gas jet stream coupled with a Novocontrol Quatro controller. Samples were placed between two flat gold-plated electrodes (10 and 20 mm in diameter) forming a parallel plate capacitor with a 0.1 mm thick Teflon spacer. Frequency sweeps were performed at a constant temperature with a stability of ±0.1 °C. BDS measurements were carried out as follows. Samples were heated up to 200 °C inside the cryostat. This temperature was held for 5 min to ensure a homogeneous filling of the capacitor and to obtain a *fully* amorphous initial state. Then, measurements started at 200 °C, cooling the samples in isothermal steps of 10 °C down to −100 °C, and subsequently heating them up to 200 °C, again in 10 °C steps. Samples were tested at different temperatures over a frequency range of 10^−1^ to 10^7^ Hz. 

## 3. Results and Discussion

### 3.1. Miscibility between PM and PVDF

The final properties of materials that are made up of more than one component can be affected by their miscibility. The Flory interaction parameter *χ*_12_ can be estimated by the following semi-empirical equation (Equation (1)) [47],

(1) $$\chi_{12}=0.34+\frac{V_{1}}{RT}\;{\left(\delta_{1}-\delta_{2}\right)}^{2}$$

where *χ*_12_ is the interaction parameter, *V*_1_ is the molar volume of the matrix component (PVDF in our case) calculated through the molar mass of the repeating unit (*M* = 64.03 g/mol) and the amorphous density (*ρ* = 1.68 g/cm^3^), in this case, *V*_1_ = 38.1 cm^3^/mol, *R* is a constant the value of which is 1.987 cal/mol K, *T* is the temperature chosen to calculate the miscibility (473 K in order to know the miscibility in the molten state), and *δ*_1_ (8.57 (cal/cm^3^)^1/2^) and *δ*_2_ (7.9 (cal/cm^3^)^1/2^) are the solubility parameters. In our case, the calculated *χ*_12_ is 0.36 at 200 °C. 

To calculate the segregation strength in the case of block copolymers, the *χ*_12_ value is multiplied by *N*, the degree of polymerization. When the value obtained is below 10, the polymers are miscible with each other; if the estimated value is between 10 and 30, there is a weak segregation; and if it is between 30 and 50, there is a medium segregation. Only when the calculated value is above of 50, it is possible to predict that there will be a strong segregation. For our samples, we have calculated that the segregation strength is 117 for the PM_23_-*b*-PVDF_77_ and 72 in the case of PM_38_-*b*-PVDF_62._ Therefore, we can expect a strong segregation in the melt for both samples.

**Figure 1:**
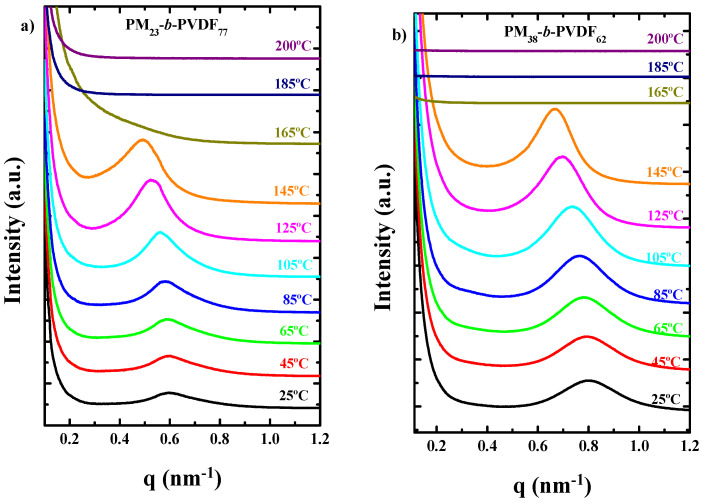
SAXS analysis at different temperatures during heating scans at 20 °C/min after a cooling process also at 20 °C/min of (**a**) PM_23_-*b*-PVDF_77_ sample and (**b**) PM_38_-*b*-PVDF_68_ sample.

Nevertheless, SAXS results do not show any evidence of phase segregation in the melt. Figure 1 shows the SAXS curves for both block copolymers at different temperatures during a heating sweep at 20 °C/min. When the copolymers are in the molten state (above 165 °C), there is not any segregation peak observed, indicating that either the electron density contrast in the melt is not enough to produce a signal or that the copolymers are either very weakly segregated or melt-mixed. The prominent SAXS peaks observed at temperatures below the melting point of PVDF are due to the average long period values of the constituent crystalline lamellae. As expected, they shift to lower q values (i.e., larger long periods) as temperature increases.

**Figure 2:**
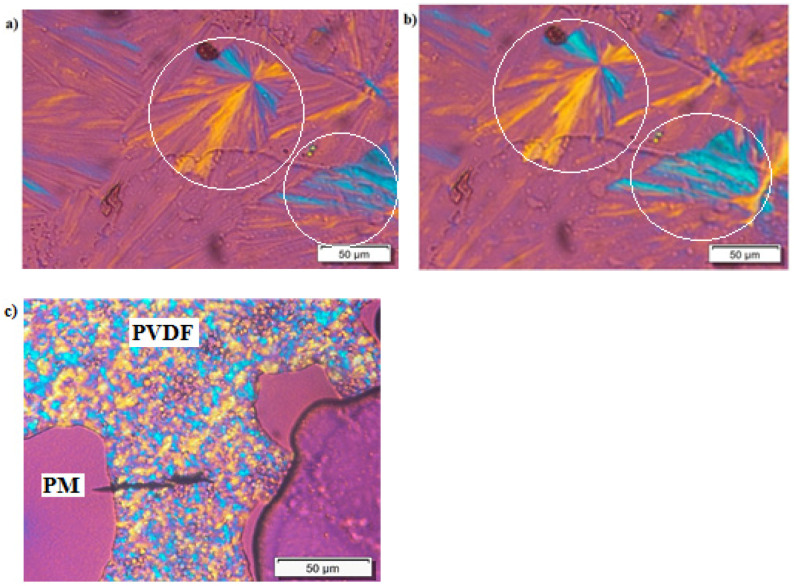
PLOM images of (**a**) PVDF block spherulites in the PM_38_-*b*-PVDF_62_ diblock copolymer sample after having been cooled at 20 °C/min to a *T* = 130 °C and (**b**) crystallization of the PM block in the PM_38_-*b*-PVDF_62_ sample after having been cooled at 20 °C/min to *T* = 25 °C. (**c**) Evident phase segregation of the PVDF and PM phases in a PM_23_PVDF_77_ blend sample after a cooling process at 20 °C/min down to *T* = 25 °C.

PLOM was used to observe the crystallization process in the different samples and to check if the segregation behavior is different between block copolymers and blends. Figure 2a shows the crystallization of PM_38_-*b*-PVDF_62_ during a cooling sweep from the melt at 20 °C/min. In a strongly segregated diblock copolymer with this composition, the expected microphase separated morphology in the melt would be that of a lamellar assembly. Additionally, if the segregation is strong, each block has to crystallize within the confined microdomain morphology produced during the phase segregation in the melt. As a result, it would be impossible to observe spherulites.

The micrograph shown in Figure 2a was taken at a temperature higher than the melting point of the PM block in the copolymer (i.e., *T* = 130 °C). The PVDF block crystallizes as spherulites in this case. This observation indicates that the diblock copolymer crystallizes either from a weakly segregated melt, from which break out leads to spherulites formation or from a melt mixed state, which can also explain the observation of spherulites. As shown in Figure 2b, when the temperature is lower than the PM block crystallization temperature (micrograph taken at 25 °C), a subtle change in the birefringence is observed. This change in birefringence has been highlighted by surrounding the most noticeable areas with a white circle. In order to quantify this, change in the transmitted light intensity during the cooling process was measured using the ImageJ software [48]. The results obtained are plotted in Figure S5 in the Supporting Information, and they conclusively show the sequential crystallization of the PVDF and PM blocks upon cooling from the melt. This change happens as the PM block crystallizes within the already formed PVDF spherulites, just within the intraspherulitic amorphous regions, as has been observed before for other block copolymer systems, such as PCL-*b*-PLLA or PEO-*b*-PCL [49,50]. The PLOM results obtained in Figure 2a,b indicate that these copolymers are either miscible or weakly segregated. These results are consistent with the lack of phase segregation observed by SAXS. 

On the other hand, Figure 2c shows the complete crystallization of both phases (PM and PVDF) in the blends after a cooling scan at 20 °C/min at *T* = 25 °C from the molten state. The phase segregation between the phases is evident. PVDF crystallizes as spherulites, and PM crystallizes in microaxialites (difficult to see in the micrograph due to their small size). This result suggests that there is evident macrophase segregation in the blends. 

**Figure 3:**
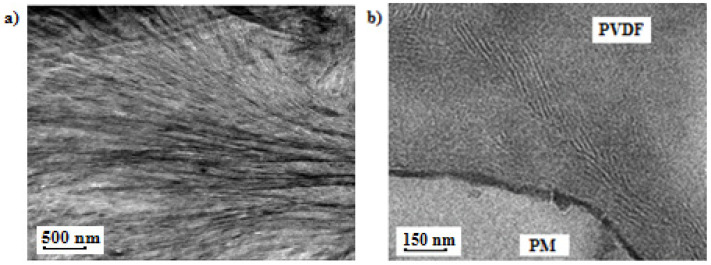
TEM images for (**a**) PM_23_-*b*-PVDF_77_ linear diblock copolymer and (**b**) PM_23_PVDF_77_ blend after cooling the samples at 20 °C/min to 25 °C.

TEM was used to see the differences in the miscibility and in the lamellar structure between the block copolymers and the blends. Figure 3 shows the TEM images for the PM_23_-*b*-PVDF_77_ diblock copolymer sample (Figure 3a) and the PM_23_PVDF_77_ blend sample (Figure 3b), respectively. Figure 3a shows a close-up region of a spherulite whose center is located to the right of the micrograph. A large number of lamellae that have grown from the right to the left of the micrograph can be observed. We were not able to distinguish the lamellae belonging to the PVDF block or to the PM block, as they seem to have similar sizes. Their co-existence without any discontinuity suggests that both blocks crystallize from a miscible melt. No signs of phase separation were observed for the block copolymer samples.

On the other hand, in Figure 3b, it is possible to observe the evident phase segregation between PVDF and PM phases in the PM_23_PVDF_77_ blend. In summary, taking into account the collected evidence by PLOM and TEM, we can conclude that the PM and PVDF samples employed here are miscible when they form diblock copolymers, but they are immiscible when they are physically blended. This aspect is important to take into account in the next sections.

### 3.2. How the Cooling Rate Affects the Crystallization of the PVDF Phase in Block Copolymers and Blends

Blends and block copolymers were studied at different cooling rates in order to observe how this parameter affects the crystallization of PVDF in both systems. The cooling rates employed were 1, 5, 20, and 60 °C/min, and the heating rate used after the cooling process was always 20 °C/min. A PVDF homopolymer was also studied for comparative purposes. 

**Figure 4:**
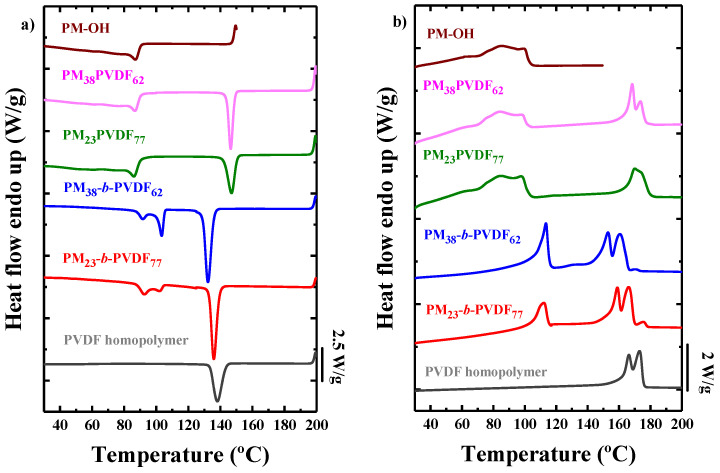
DSC scans of the blends, the diblock copolymers, and homopolymers samples. (**a**) Cooling curves at 20 °C/min and (**b**) heating curves at 20 °C/min after the previous cooling process.

Figure 4a shows the DSC cooling scans at 20 °C/min of the PVDF homopolymer, the PM homopolymer (PM-OH), the two different diblock copolymers, and their respective blends at the same composition. The crystallization (Figure 4a) peaks located at higher temperatures correspond to the PVDF component. In the blends, the PVDF component crystallizes at higher temperatures than the PVDF homopolymer (which is one of the components used to formulate the blend). This corresponds to a nucleating effect of the molten PM-OH phase, which can be explained by a transference of impurities from the PM phase to the PVDF phase during blending, as already described for other systems [51,52,53]. On the other hand, the PVDF blocks in the diblock copolymers have lower *T_c_* values than the PVDF homopolymer sample, a possible sign of miscibility between the blocks. The other crystallization peak, at lower temperatures, corresponds to the PM blocks. In this case, the crystallization of the PM in the diblock copolymers is bimodal and occurs at higher temperatures than those observed for the blends and for the PM homopolymer. This higher crystallization temperature could be related to a nucleating effect of the PVDF block crystals.

The DSC subsequent DSC heating curves taken at 20 °C/min are plotted in Figure 4b and show that the melting peak that corresponds to the PM crystalline phase shows up at lower temperatures than that one observed for the PVDF. It is clear that the blends are totally immiscible, and the melting points of the PM phase (which shows a bimodal character) in the blends are very similar and located at the same temperatures as in the PM homopolymer. On the other hand, in the block copolymers, the PM block melting peak is a monomodal sharp endotherm that peaks at significantly higher values than that of the PM homopolymer or the PM phase in the blends. Regarding the melting peaks associated to the PVDF phases in the blends, these are located in the same temperature range as those of the PVDF homopolymer, once again suggesting that PM and PVDF are immiscible. In summary, due to the phase segregation encountered in the blends, the melting peaks of the blends correspond to those observed for their homopolymers in the same temperature range.

For the PVDF phase, melting is characterized by two main peaks. Due to the polymorphism observed in PVDF, different phases can form in the same sample [54]. In the case of the diblock copolymers, even a third minor peak appears at higher temperatures. This peak could be either a third crystalline phase or the result of a crystal reorganization that occurred during the heating process. The first melting peak in PVDF usually corresponds to the less stable, ferroelectric β-phase, and the second melting peak, to the paraelectric α-phase [30]. 

**Figure 5:**
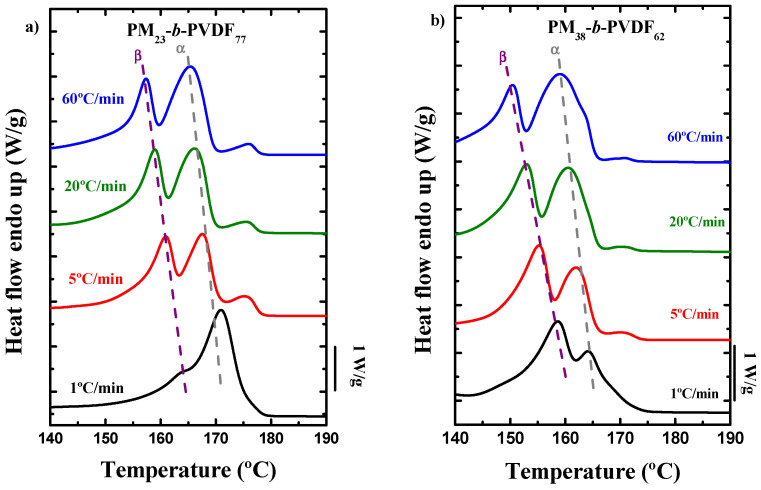

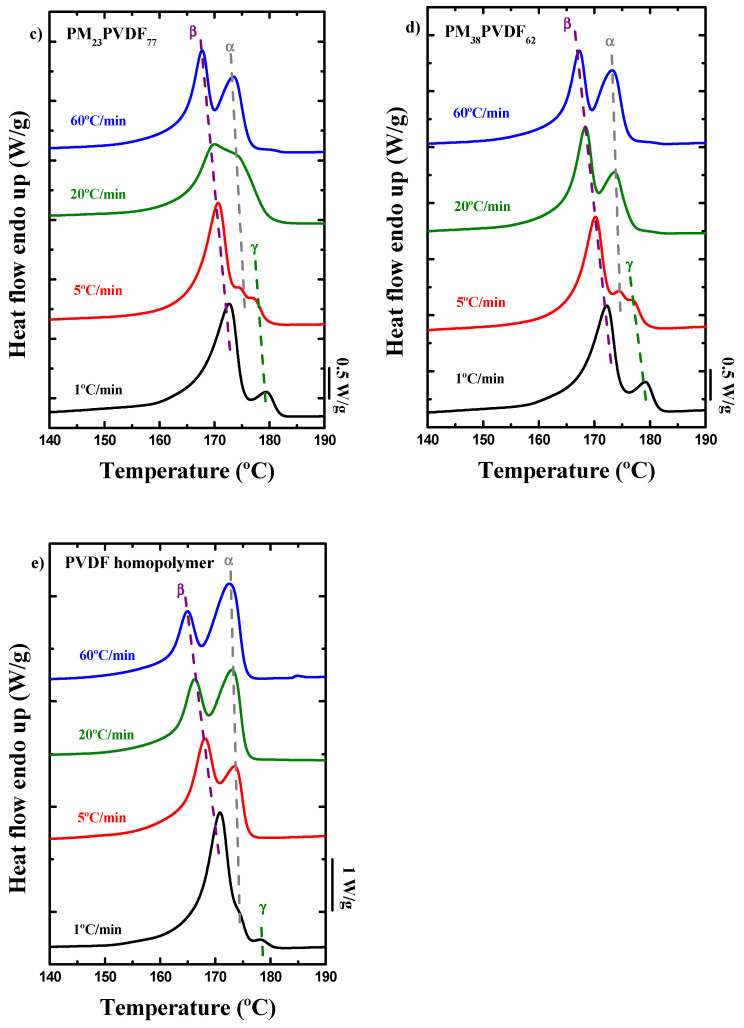
DSC heating scans for PVDF after different cooling rates were used: (**a**) PM_23_-*b*-PVDF_77_ and (**b**) PM_38_-*b*-PVDF_62_ block copolymers, (**c**) PM_23_PVDF_77_, (**d**) PM_38_PVDF_62_, and (**e**) PVDF homopolymer samples.

Figure 5 shows the comparison of the DSC heating scans of the samples (all performed at 20 °C/min) in the PVDF melting range obtained after using different cooling rates. The PM_23_-*b*-PVDF_77_ diblock copolymer (Figure 5a) shows three melting peaks at all the cooling rates studied, except at 1 °C/min, where only one main peak with a low temperature shoulder is observed. The third peak that can be observed at around 175 °C seems to be related to a crystal reorganization process, and Figure 5a shows that it does not depend on the cooling rate used (except for the experiment performed at 1 °C/min). The height and the area of the other two peaks seem to remain constant at all the cooling rates except at 1 °C/min, where the behavior of the subsequent melting curve is completely different. First, there is not a third peak, and second, the first peak, probably the β-phase peak, has almost disappeared, so at 1 °C/min, the α-phase peak is promoted. This is a common behavior reported in the literature for the PVDF: at low cooling rates, the formation of the most stable phase is promoted [55,56].

The second diblock copolymer (Figure 5b), PM_38_-*b*-PVDF_62_, shows different behavior. At high cooling rates, the α-phase peak is larger than the β-phase peak, but when the cooling rate is decreased, the α-phase peak also decreases, and the β-phase peak is the majority phase in the copolymer. For instance, at 1 °C/min, the promotion of the β-phase is evident. The crystallization behavior of the PVDF at 1 °C/min is completely different than the behavior shown by the PM_23_-*b*-PVDF_77_ copolymer: the formation of the less stable phase is promoted in this case. 

On the other hand, both PM/PVDF blends exhibit similar behavior (Figure 5c,d). In this case, it seems that the amount of PM in the blend has no effect on the crystallization of the PVDF phase. The formation of the β-phase is always promoted in the blends, even at high cooling rates, where it coexists with the α-phase. When the cooling rate is decreased (5 °C/min), the α-phase almost disappears, and a new high temperature peak appears, which is associated to a different crystalline phase that is more stable than the last two ones explained. It has been reported in the literature that at these high temperatures (higher than 175 °C) the γ-phase, which is also polar, crystallizes [57,58]. When samples are cooled at 1 °C/min, the α-phase peak completely disappears, and the β-phase and the γ-phase coexist. For comparative purposes, a PVDF homopolymer was also studied at different cooling rates (Figure 5e). As can be seen at high cooling rates, the α-phase and the β-phase coexist; however, when the cooling rate is decreased, the PVDF tends to crystallize preferentially in the β-phase. At 1 °C/min, the three crystalline phases mentioned above coexist, and the β-phase is the main crystalline phase. A small shoulder at high temperatures corresponds to the α-phase, and finally, the new stable melting peak appears, which probably corresponds to the previously mentioned γ-phase. All the calorimetric parameters obtained by DSC are listed in Table 2.

**Figure 6:**
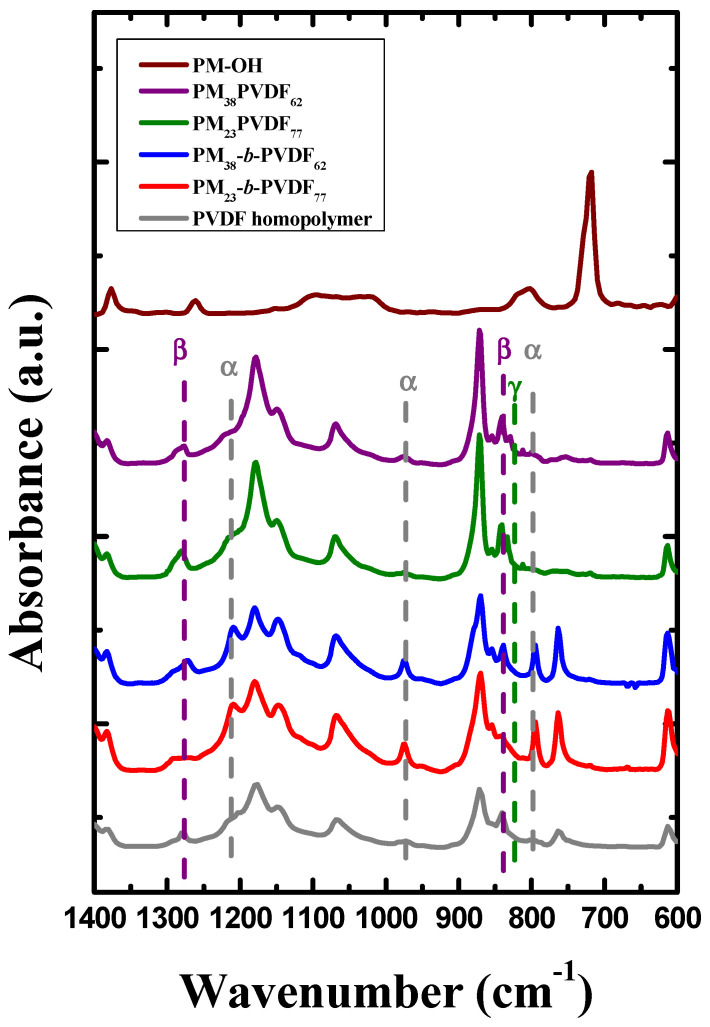
Sections of FTIR spectra of PM-OH, PVDF homopolymer, PM_23_-*b*-PVDF_77_, PM_38_-*b*-PVDF_62_, PM_23_PVDF_77_, and PM_38_PVDF_62_ samples after a cooling sweep at 1 °C/min. The grey dashed line shows the bands for the α-phase; the purple dashed line is for the β-phase, and the green dashed line corresponds to the γ-phase.

DSC heating scans performed after cooling the samples at 1 °C/min show that the crystalline phase obtained depends on the sample and the origin of the sample. Samples cooled at 1 °C/min were analyzed by FTIR to verify which phases the PVDF block crystallizes in. Figure 6 shows the FTIR results for the PM homopolymer, the PVDF homopolymer, both diblock copolymers, and both blends, at room temperature after the samples were cooled from the melt at 1 °C/min. The wavenumber range studied was 1400–600 cm^−1^, which is where the most useful information for PVDF can be observed. There is a large band located at 720 cm^−1^ and a smaller one at 1377 cm^−1^, where the main characteristic bands for the PM polymer are observed [59]. There is also a weak band located at 801 cm^−1^. We can observe that the main peaks perceived for PM do not overlap with the main bands associated with PVDF. 

When the crystallization of the PVDF homopolymer happens at a low cooling rate, three very weak bands can be seen at 1214, 976, and 796 cm^−1^, which correspond to the α-phase. This means that the formation of the α-phase is not really promoted in the homopolymer. Moreover, there are two additional, more intense, main bands, at 1275 and 840 cm^−1^, which correspond to the crystalline β-phase. This means that, surprisingly, the PVDF homopolymer is able to crystalize in the ferroelectric β-phase when the polymer is crystallized slowly from the melt. 

The spectra for both diblock copolymers show bands for the crystalline α-phase and β-phase. The PM_23_-*b*-PVDF_77_ shows only one small band located at 1278 cm^−1^, corresponding to the β-phase, but there is not any band at 840 cm^−1^. This indicates the presence of a small amount of β-phase in the copolymer. In addition, the FTIR spectrum of this sample clearly shows the bands corresponding to the α-phase, which indicates that the crystallization observed at 1 °C/min corresponds mainly to the paraelectric α-phase, which confirms the DSC results. 

On the other hand, the spectrum of the PM_38_-*b*-PVDF_62_ sample shows the α-crystals bands mentioned before and the band located at 1278 cm^−1^ that corresponds to the β-phase. The FTIR analysis of this diblock copolymer demonstrates that the α-phase and the β-phase coexist simultaneously after samples have been cooled at 1 °C/min. Again, this behavior confirms the DSC results: at low cooling rates, the formation of the β-phase is promoted, but the α-phase remains present. 

The FTIR spectra for the two blends (Figure 6) show the two main bands corresponding to the β-phase plus a new band located at 811 cm^−1^, which corresponds to the γ-phase crystals [60]. All the characteristic bands for PM and PVDF are shown in Table 3.

**Figure 7:**
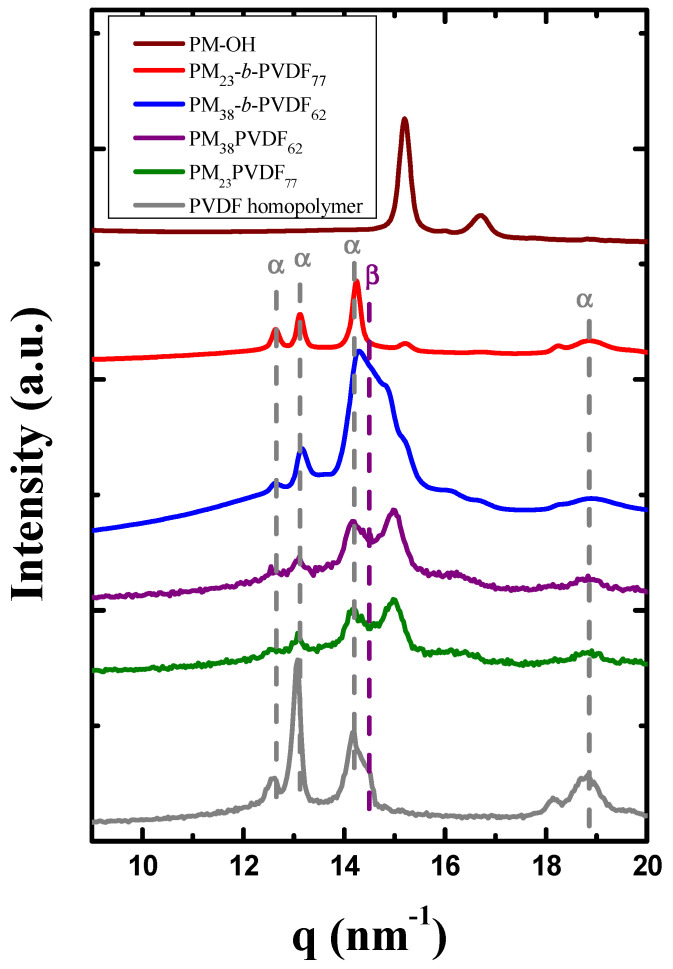
WAXS diffraction patterns of PM-OH, PVDF homopolymer, both blends, and both block copolymers at room temperature after a crystallization process at 1 °C/min. The grey dashed lines indicate the peaks associated to the α-phase, and the purple dashed line indicates the peak of the β-phase.

WAXS experiments were performed to investigate what phases crystallized during the cooling process at 1 °C/min from the molten state (Figure 7). The main reflections for the PM are located at 15.2 and 16.7 nm^−1^ as can be seen in the pattern of the PM-OH sample. PM crystallizes in an orthorhombic unit cell with parameters a = 0.742 nm, b = 0.495 nm, c = 0.255 nm, and β = 90°, with a *P-D*_2*h*_ space group [63,64]. The crystallographic planes for these peaks are (110) and (200), respectively [65,66]. 

PVDF has different crystalline phases, which appear as WAXS reflections at different *q*-values (see Figure 7). The peaks that are located at *q*-values of 12.6, 13.1, 14.2, and 18.9 nm^−1^ correspond to the crystalline α-phase, and the reflections of this paraelectric phase have the following crystallographic planes: (100), (020), (110), and (120/021) [67,68,69]. The α-phase of PVDF is characterized by a pseudo-orthorhombic unit cell with a = 0.496 nm, b = 0.964 nm, c = 0.462 nm, and β = 90° and has a *P2/C* space group [70,71]. In our case, these reflections appear for the diblock copolymers, the blends, and the homopolymer. These reflexions are more intense in the homopolymer and in the PM_23_-*b*-PVDF_77_ than in the other samples. Based on this result and the FTIR spectra, we can conclude that during the crystallization of the PM_23_-*b*-PVDF_77_ the formation of the α-phase is always promoted at low cooling rates.

However, apart from the characteristic peaks of the α-phase, the other samples containing PVDF display one extra peak or shoulder in their patterns at 13.5 nm^−1^ (Figure 7). This new reflection corresponds to the crystallization of the β-phase, which has the (200/110) crystal plane [18]. The β-phase of PVDF is characterized by an orthorhombic unit cell, which has a *Cm2m* space group and the following dimensions: a = 0.847 nm, b = 0.490 nm, and c = 0.256 nm [72]. The presence of this peak is in agreement with the results obtained before in the DSC analysis, which suggests that the formation of the β-phase is promoted in samples that were previously cooled at 1 °C/min and coexists with a small amount of crystalline α-phase. It seems that the amount of PM in the diblock copolymer can affect the PVDF crystallization in order to promote the desired β-phase.

### 3.3. Dielectric Spectroscopy Studies in PVDF and Its Copolymers

**Figure 8:**
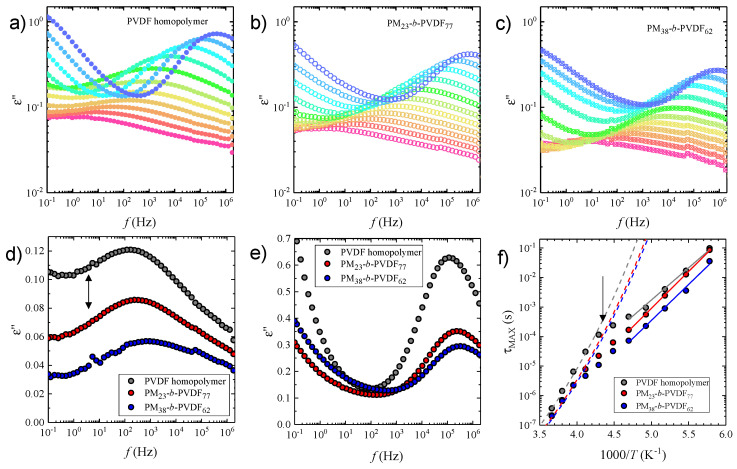
Dielectric spectra (imaginary part of the complex dielectric permittivity as a function of the frequency) for (**a**) PVDF homopolymer, (**b**) PM_23_-*b*-PVDF_77_, (**c**) PM_38_-*b*-PVDF_62_, as well as dielectric relaxations of the studied samples at (**d**) −70 °C and (**e**) −10 °C and (**f**) relaxation map of the studied samples.

Figure 8 shows the BDS results for PVDF and its copolymers with PM. In particular, Figure 8a–c displays dielectric spectra: the imaginary part of the complex dielectric permittivity as a function of the frequency. The data presented correspond to the one collected by isotherms from −100 to 0 °C in steps of 10 °C (measured on heating). The corresponding experiments on cooling were nearly indistinguishable. In general, the relaxation processes were characterized by a single maximum, which shifted towards higher frequencies and increased in intensity as the temperature was increased. 

At low temperatures (−100 °C to −60 °C), a weak and broad peak was observed for all samples, although with different characteristics. PVDF displayed the highest intensity peaks, reaching *ε*″ values of around 0.1. In the case of the diblock copolymers, the intensity of the relaxations decreased with PM content. We also observed that, as PM content increased, the relaxation peaks maxima shifted towards higher frequencies. As an example, Figure 8d shows the dielectric relaxations of the samples at −70 °C. In addition to the differences already discussed, PVDF displayed a pronounced asymmetry towards low frequencies (black arrow in Figure 8d). However, the relative intensity of this low-frequency signal decreased for the samples containing PM blocks. 

Comparing with previous literature reports, and taking into consideration the intensity and position of the peaks, we were able to assign the low-temperature process to the local β-relaxation of PVDF related to local motions of polar groups in the polymer [73,74,75,76]. As the temperature was further increased (*T* > −60 °C), the relaxation peaks suffered important changes. In all cases, as the maxima moved towards higher frequencies, the peaks were narrower and showed a dramatic intensity increase. These changes in the dielectric relaxation occurred at temperatures close to the glass transition of PVDF (−43 to −23 °C) [77]. Thus, we could relate the changes to the α-relaxation of the PVDF. This relaxation process is related to the segmental motion of the PVDF polymer chain taking place at temperatures above the glass transition (*T*_g_), as widely reported [73,74,75,78,79]. Please notice that our experimental results showed a continuous change in the dielectric spectra, going from the β- to α-relaxation, instead of separated peaks observed in previous works [73,74,75,78,79]. Nonetheless, although in our current work the α-relaxation peak could not be well resolved at low frequencies, the data showed an increased broadness at *T* = −50 to −40 °C. The peak was better resolved in the PVDF sample than in PM-*b*-PVDF copolymers, which indicates that the PVDF segmental relaxation was affected by the presence of PM units. In fact, in the −50–0 °C temperature range, PM-*b*-PVDF copolymers showed lower segmental relaxation intensities and slightly faster dynamics compared to the PVDF. Figure 8e presents a comparison of the datasets at −10 °C where this evidence can be observed. 

Figure 8f shows the relaxation map of the samples. The relaxation time (*τ*_MAX_) was calculated from the maxima of the dielectric relaxation peaks. In all cases, we observed two trends in the temperature dependence of relaxation times. At low temperatures (−100 ≤ *T* (°C) ≤ −60), the relaxation times followed an Arrhenius behavior, as described by [80]:

(2) $$\tau_{\mathrm{MAX}}=\tau_{0}\exp\left[\frac{E_{A}}{kT}\right]$$

where $E_{A}$ is the activation energy, k is Boltzmann’s constant, and $\tau_{0}$ a pre-exponential factor. The obtained results are shown in Figure 8f as continuous lines and are summarized in Table 4. For PVDF, we found $E_{A}$ = 42 kJ/mol, which increased slightly for the PM-*b*-PVDF systems (~48 kJ/mol). These values are quite similar to the one reported before by Sy and Mijovic (~43 kJ/mol) [73] for the local relaxation of PVDF, while slightly lower than that observed by Linares and collaborators (~60 kJ/mol) [74].

At temperatures above −60 °C, the relaxation times of the samples showed a deviation from the low-temperature Arrhenius trend. In all the studied samples, a sort of “kink” appeared at temperatures around −50 to −60 °C (see arrow in Figure 8f). We related these changes to the effect of the segmental relaxation of PVDF on the relaxation times. We also observed that the kink’s intensity was reduced in the block copolymer as the PM content increased. These sorts of trends, or anomalies, have been reported before for PVDF-based systems. For example, Sy and Mijovic observed a similar behavior in local motions of semicrystalline PVDF/PMMA blends [73]. In that work, the temperature dependence of the relaxation times of PVDF/PMMA blends was described as a gradual crossover from local to segmental motions, which was clearly different from an α-β merging. The 90/10 PVDF/PMMA showed the most pronounced kink, which decreased as the PMMA content increased. However, the neat PVDF did not show this signature. Martínez-Tong et al. [81] also observed a continuous transition in the dielectric relaxation map of a PVDF copolymer with trifluoroethylene (PVDF-TrFE), with a VDF mol content of 76%. In that work, the authors observed a crossover from the segmental relaxation to the ferroelectric-paraelectric relaxation of the polymer. Just at the transition temperatures (~47–57 °C), a small kink can be detected in the relaxation plot. Finally, very recently, Napolitano and collaborators observed an anomalous behavior in the local relaxation of PVDF copolymers with hexafluoropropylene (HFP) [76]. In their work, the dielectric relaxation experiments showed that, in the vicinity of *T*_g_, the PVDF-HFP copolymers displayed a so-called “anomalous minimum” in the local relaxation. The authors related their findings to the bonds formed by fluorine entities, similar to those observed in propylene glycol systems. Moreover, the authors also observed that the anomalous process weakened when the PVDF-HFP samples were prepared as ultrathin polymer films. This nanoconfinement-induced reduction in the anomaly was explained by means of the minimal model and related to an asymmetry in the well potential describing the molecular motion. In this work, we observed that PM-*b*-PVDF samples showed a reduction of the observed kink, whose intensity decreased as PM content was increased. This could indicate that the PM block is inducing local confinement effects on the samples. 

Finally, we attempted to model the data points in the −50–0 °C temperature range using the Vogel–Fulcher–Tamman (VFT) equation, described by [80]:

(3) $$\tau_{\mathrm{MAX}}=\tau_{\infty }\exp\left[\frac{DT_{\mathrm{VFT}}}{T-T_{\mathrm{VFT}}}\right]$$

where $\tau_{\infty }$ is a pre-exponential factor, D is a dimensionless parameter related to the dynamic fragility [82], and $T_{\mathrm{VFT}}$ the Vogel temperature. The results obtained are summarized in Table 4 and the fits are shown in Figure 8f by dashed lines. We highlight that the value of $\tau_{\infty }$ was set at 10^−14^ s, based on the discussion of Angell [82,83]. For all samples, we obtained a *D* = 21, indicating a small deviation from an Arrhenius process. This value was slightly larger than the ones reported before (*D* = 12–15) for PVDF [73,79]. However, it was fairly comparable to the one obtained by Martínez-Tong and collaborators for the PVDF-TrFE copolymer (*D* = 21.6). Finally, we were able to predict the dynamic glass transition temperature ($T_{g-\mathrm{BDS}}$) of the samples in our study from the VFT fit. This parameter was defined as the temperature where the segmental relaxation time reached 100 s. The results obtained, shown in Table 4, allowed to determine a $T_{g-\mathrm{BDS}}$ = −80 °C for PVDF. This value decreased for the PM-*b*-PVDF samples with increasing PM content, which was in line with the faster dynamics observed. The $T_{g-\mathrm{BDS}}$ obtained were lower than the usual ones reported for PVDF by different methods ($T_{g}$ = −63 to 23 °C) [84,85]. However, we emphasize that both the PVDF and PM-b-PVDF copolymers have low molecular weights (6–8 kDa), which would explain the obtained results. In addition, we should take into account that, in semicrystalline polymers, the dynamics in the more amorphous environments dominate the dielectric relaxation peak frequency position [86].

### 3.4. How the Isothermal Crystallization Affects PVDF Blends and Block Copolymer Samples

**Figure 9:**
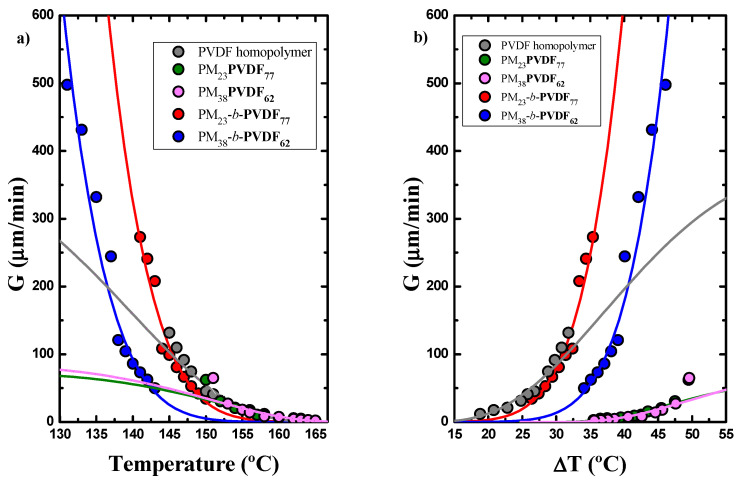
(**a**) Spherulitic growth rates determined by PLOM for homopolymer PVDF, the PVDF block of the diblock copolymers, and the PVDF phase within the blends studied and (**b**) spherulitic growth rates as a function of supercooling. The solid lines are the fits to the Lauritzen–Hoffman (LH) theory.

Figure 9 shows the spherulitic growth rate of PVDF, its copolymers and the prepared blends as a function of the isothermal crystallization temperature. The high nucleation density observed in the blends only allow us to measure spherulites at relatively high crystallization temperatures. Experiments were performed by cooling the samples from the melt to a chosen crystallization temperature in the range from 131 to 164 °C. Spherulitic growth rates for each sample, *G* (μm/min), were determined at different crystallization temperatures from the slope of radius versus time plots (which were always linear).

Figure 9a shows the spherulitic growth rate *G* (μm/min) as a function of *T_c_*. As can be seen, the growth rate is faster in the copolymers than in the blends and the homopolymer sample in the low temperature range. However, the comparison is difficult, as the crystallization ranges of the sample do not overlap. *G* dramatically decreases when the PVDF is blended with PM. The supercooling required for crystallization increases when the PVDF is blended with PM, as a result of the change in the equilibrium melting temperature. When *G* is plotted as a function of supercooling (*ΔT* = *T_m_*^0^ − *T_c_*), using the equilibrium melting temperatures (*T_m_*^0^) determined by the Hoffman–Weeks method, in Figure 9b, the curves are now shifted along the x-axis reducing the differences between the overall crystallization curves versus *T_c_*. In this representation as a function of supercooling, it is easier to observe the above mentioned trends. 

It is unexpected that the growth rate (Figure 9b) of the PVDF component decreases in the blends as compared to the neat PVDF. One possible explanation could be that even though the blends are immiscible (as indicated by the DSC results), the molten PM-OH is capable of interacting with the PVDF (though the OH group) reducing the PVDF diffusion to the growth front.

In the diblock copolymers case, the growth rate of the PVDF block decreases as the PM content in the copolymer decreases. It can also be noted that the temperature dependence of the growth rate between the neat PVDF homopolymer and the PVDF blocks in the diblock copolymers is very different. This is easily captured by the Lauritzen and Hoffman fits, which are represented as solid lines in Figure 9.

Isothermal crystallization experiments were performed by DSC to determine the overall crystallization rate of the samples (which include both nucleation and growth contributions). Differences in the PVDF polymorphism and its crystallization kinetics were observed depending on the structural forms of the respective samples. The Avrami theory and the Lauritzen and Hoffman theory were employed [87,88] to describe the primary crystallization process in polymers and to plot several kinetic crystallization parameters as a function of the crystallization temperature.

**Figure 10:**
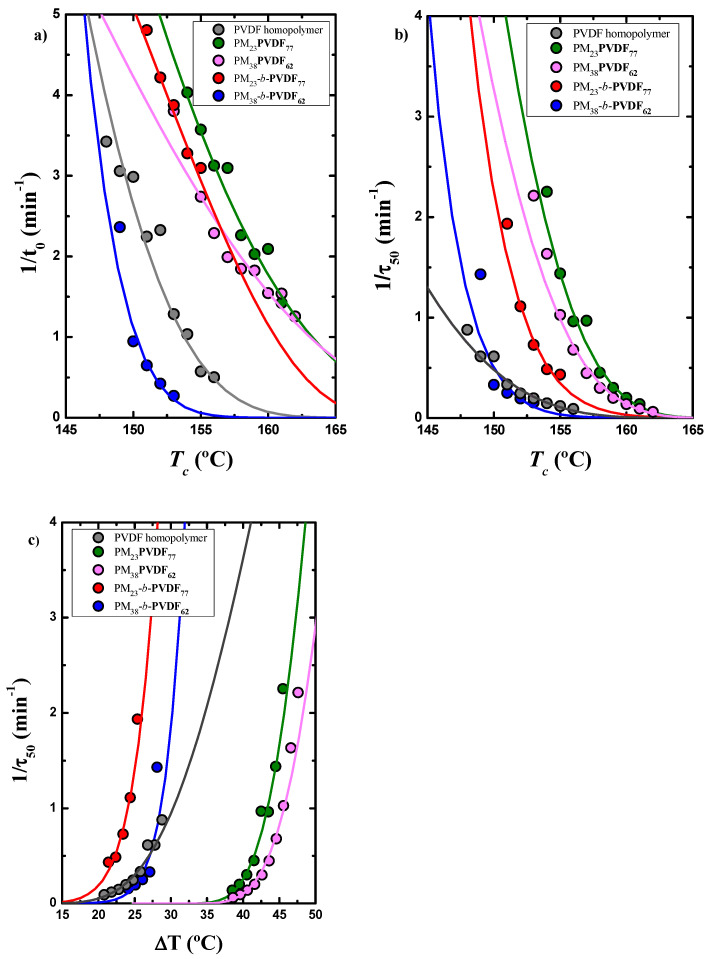
(**a**) 1/t_0_ as a function of crystallization temperature and inverse of half-crystallization time for the PVDF component of all samples shown as a function of (**b**) *T_c_* and (**c**) Δ*T* for all the PVDF samples measured by DSC. The solid lines are the fits to the Lauritzen–Hoffman (LH) theory.

Figure 10a shows the inverse of the induction time (*t*_0_) versus the isothermal crystallization temperature (*T_c_*) for the different PVDF samples. The induction time is equivalent to the primary nucleation time before any crystallization is detected by the DSC. The inverse of the induction time is proportional to the primary nucleation rate of the PVDF components in the different samples. The nucleation rate depends on the composition and the nature of the samples. The nucleation rate of the PVDF block within the PM_38_-*b*-PVDF_62_ sample is faster than in the homopolymer sample, while in the blend, the PVDF phase has a slower nucleation rate. 

Figure 10b shows the inverse of the half crystallization rate (*τ*_50%_) versus the isothermal crystallization temperature (*T_c_*). The 1/*τ*_50%_ value is the inverse of the time needed to achieve the 50% of the total transformation to the semi-crystalline state during the isothermal crystallization process and represents an experimental measure of the overall crystallization rate, which includes both growth and nucleation contributions.

Figure 10b reflects a combined trend of the observed nucleation behavior (Figure 9a) and the spherulitic growth behavior (Figure 9a). Both the PVDF homopolymer and the PM_23_PVDF_77_ blend exbibit the lowest overall crystallization rates. However, as in the overall crystallization, both nucleation and spherulitic growth rate contribute; in this case, 1/*τ*_50%_ does not decrease as dramatically as *G* for the rest of the materials. Therefore, the changes in nucleation density strongly affect the overall crystallization rates determined by DSC in these PVDF-based blend samples. Figure 10c shows these results when they are plotted against the supercooling (*ΔT*) and the curves are shifted in the x-axis standardizing the differences in crystallization temperature exhibited by the different samples.

The Avrami theory is a useful tool to fit the overall crystallization kinetics of polymers during the primary crystallization regime [89,90,91]. The Avrami theory is given by the following equation:

(4) $$1-V_{c}\left(t-t_{0}\right)=exp\left(-k{\left(t-t_{0}\right)}^{n}\right)$$

where *V_c_* is the relative volumetric transformed fraction, *t* is the time of the experiment, *t*_0_ is the induction time before the crystals start to grow, *k* is the overall crystallization rate constant, and *n* is the Avrami index, which is related to the time dependence of the nucleation and the crystal growth geometry.

**Figure 11:**
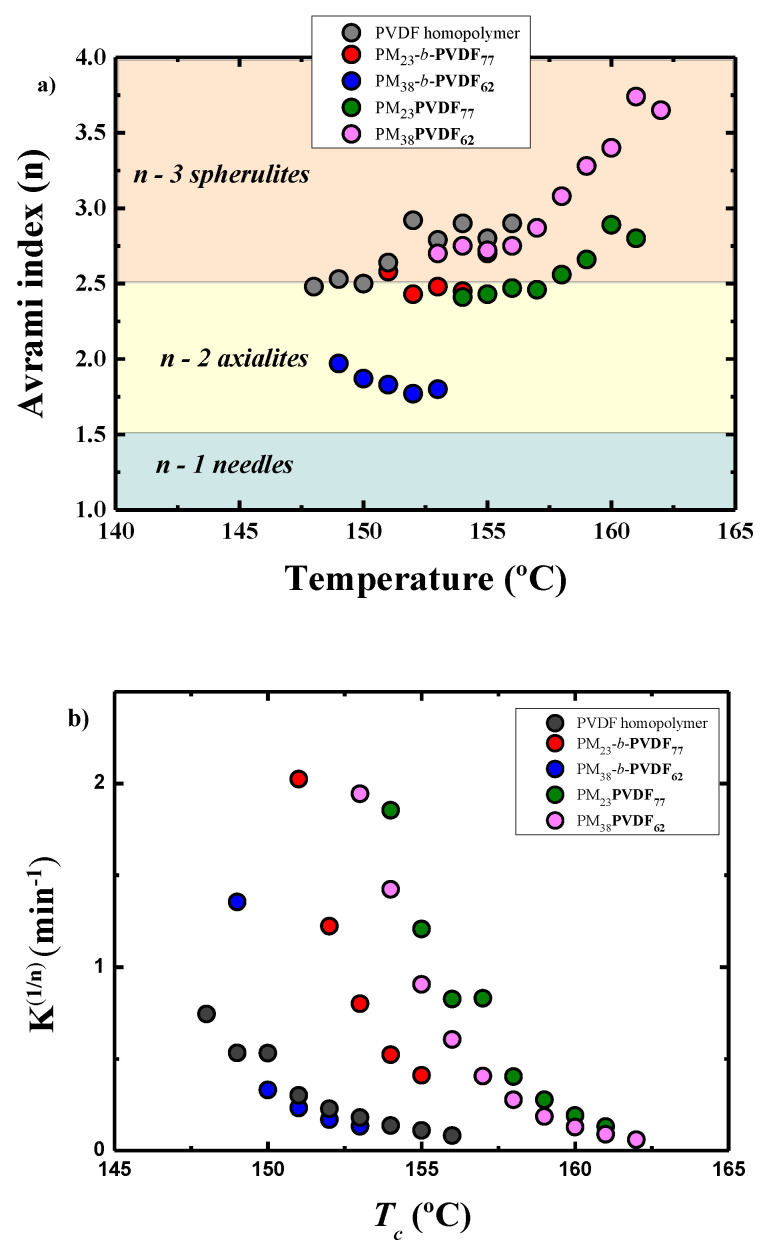
(**a**) PVDF Avrami index values for all the temperatures used in the isothermal crystallization and (**b**) isothermal crystallization rate obtained by the Avrami model.

By applying the Avrami equation to the isothermal crystallization curves at each chosen crystallization temperature, it is possible to calculate the Avrami index (*n*), but it is only possible when the crystallization starts at the isothermal temperature selected and not during the cooling, as happened in the case of the PM. Figure 11a shows all the *n* values for the crystallization of the PVDF component in all the samples studied during this work. Usually, for polymers, *n* is between 1.5 and 4. When this value is higher than 2.4, the crystals of the polymer grow as spherulites. In our case, all the samples have an *n* value higher than 2.5 with the exception of the PM_38_-*b*-PVDF_62 _sample. For the samples with an *n* value below 2.5, crystals grow in 2D, forming axialites. Figure 11b shows the evolution of the *k*^1/*n*^ value at different crystallization temperatures, and these values are proportional to the overall crystallization rate. The comparison between Figure 9b and Figure 11b demonstrates that the theoretical results obtained through the Avrami theory are really close to the experimental results obtained using the Lauritzen and Hoffman method as the trends in the data are similar(1/*τ*_50%_).

The value of the equilibrium melting temperature of each sample was calculated using the Hoffman–Weeks method; see the Supporting Information. The values obtained are listed in Table S2 in the Supporting Information.

**Figure 12:**
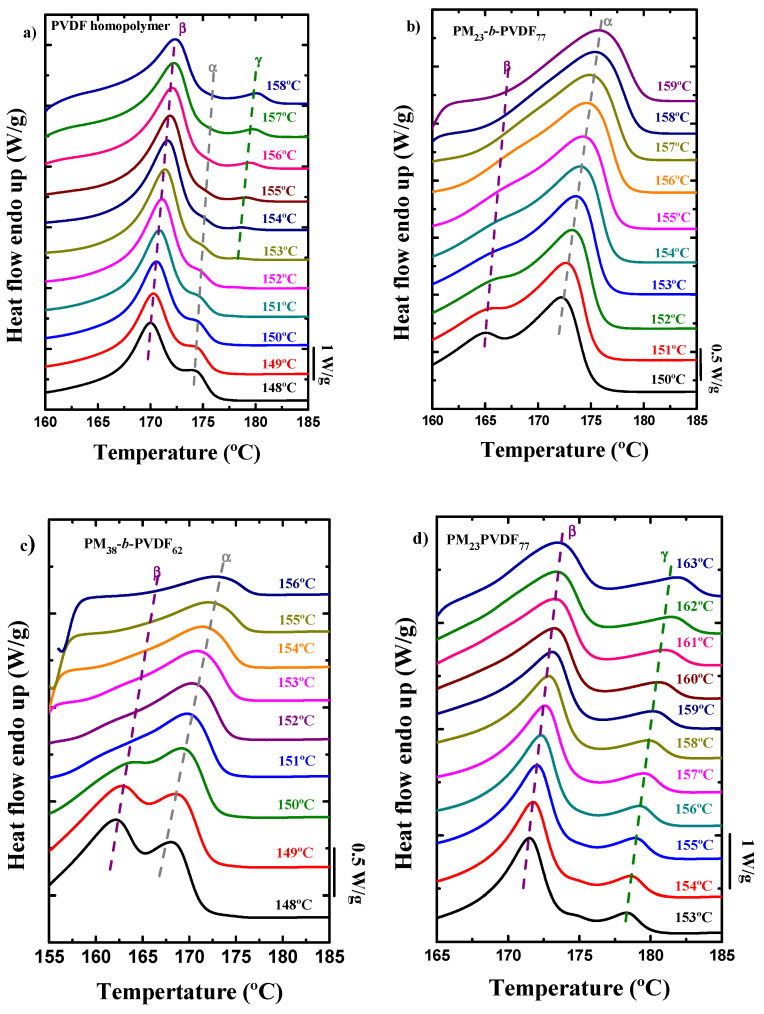

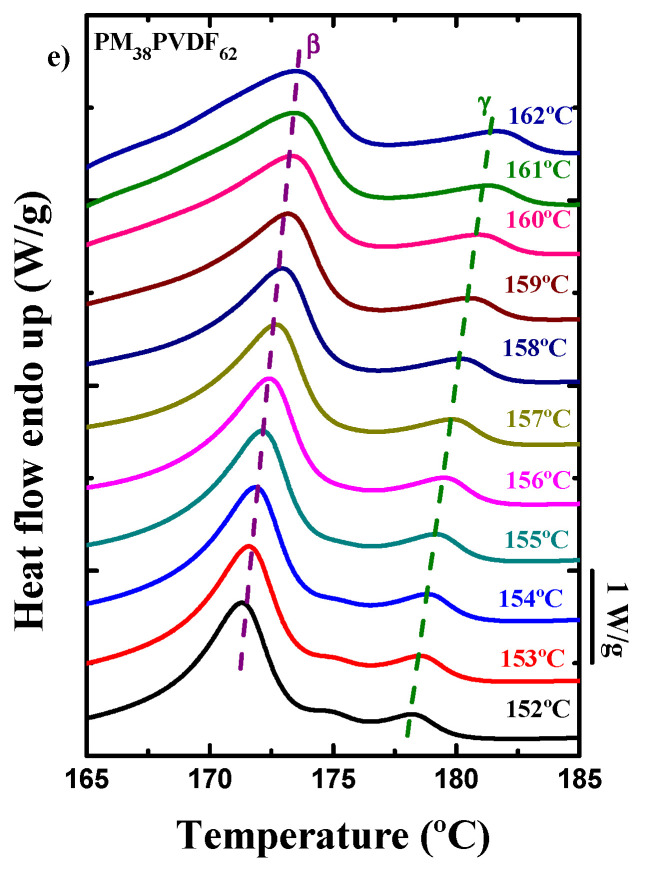
DSC PVDF melting curves after the isothermal crystallization at different temperatures of (**a**) PVDF homopolymer, (**b**) PM_23_-*b*-PVDF_77_, (**c**) PM_38_-*b*-PVDF_62_, (**d**) PM_23_PVDF_77_, and (**e**) PM_38_PVDF_62_ samples.

The analysis of the heating curves after the isothermal crystallization processes may allow us to know how the PVDF crystallizes and which crystalline phase is obtained after these procedures. Figure 12 shows all the melting curves for the PVDF component in each sample at all the isothermal crystallization temperatures studied. The *T_c_* selected through the *T_c,_*_min_ method are similar for the block copolymers and the homopolymer sample, while the blends have higher *T_c_* values. 

The PVDF homopolymer (Figure 12a) has two melting peaks when the isothermal crystallization temperature used was low: one main peak at low temperatures and another small peak at higher temperatures. The main peak corresponds to the β-phase, and the second peak to the α-phase. When the crystallization temperature increases, the peak from the α-phase starts decreasing until it disappears and a new peak appears at even higher temperatures. This new peak corresponds to the crystalline γ-phase. This means that the PVDF low molecular weight homopolymer sample can crystallize in all ferroelectric phases when submitted to low cooling rates and also during an isothermal process at high crystallization temperatures.

The behavior of the PVDF block in the diblock copolymers (Figure 12b,c) is completely different from the homopolymer sample. In this case, only two melting peaks are observed when the isothermal crystallization temperature used was low. In the case of the PM_23_-*b*-PVDF_77_ sample, the main peak is observed at higher temperatures. When the crystallization temperature increases, the first peak tends to disappear and only the main peak, which belongs to the α-phase, remains. 

For the PM_38_-*b*-PVDF_62_ sample, at low crystallization temperatures, the first melting peak is promoted (β phase), but as the isothermal crystallization temperature is increased, the size of this peak starts to decrease, and at high crystallization temperatures, only one peak is observed, which also corresponds to the α-phase. 

Both PM/PVDF blends (Figure 12d,e) have similar melting curves regardless of the PM content. Both blends show three peaks at low isothermal crystallization temperatures: The largest one is located at low temperatures and corresponds to the β-phase; then, there is a shoulder at about 175 °C, which is the melting peak of the α-phase, and finally, the last one at higher temperatures is the melting peak of the γ-phase. When the crystallization temperature is increased, only the shoulder of the α-phase disappears, while both ferroelectric phases remain. 

As during isothermal crystallization, the PVDF component develops a complex polymorphic structure that changes with crystallization temperature; this helps to explain the complex trends observed in the growth kinetics (Figure 9), nucleation rate (Figure 10a), and overall crystallization rate (Figure 10c). 

## 4. Conclusions

The complex crystallization of PVDF was found to depend on the nature of its chemical environment. We found significant differences in crystallization and polymorphic structure depending on whether the PVDF was a homopolymer (the homopolymer of the diblock copolymers), present as a block in the studied diblock copolymers, and present as a phase in the blends. The crystallization conditions were also found to dramatically affect the number and amount of the polymorphic crystalline phases produced.

DSC, PLOM, and TEM results clearly indicated that the blends prepared here are immiscible and phase segregate. On the other hand, the linear diblock copolymers crystallize from a mixed melt or very weakly segregated melt according to SAXS, TEM, and PLOM.

We were able to clearly identify the different crystalline phases form by the PVDF component in the different samples examined (i.e., α, β, and γ phases) by DSC, FTIR, and WAXS. Their number and content varied depending on sample composition, cooling rate employed, or isothermal crystallization temperature used during isothermal crystallization tests.

The BDS results indicated that the PVDF block in the copolymers has lower *T_g_* values than the homopolymer, which was in line with the faster chain dynamics observed in them. The spherulitic growth rates, nucleation rates, and overall crystallization rates were determined, and different values were obtained depending on the sample. This is not surprising considering that the melting after isothermal crystallization revealed that the polymorphic structure of each sample varied during isothermal crystallization. 

## Figures and Tables

**Table 1:** Principal characteristics of all samples employed during this work. The subscripts indicate the wt% of each block.

Sample	Topology	*M_n_* (g/mol) ^a^	*M_n_* PM (g/mol*)* ^a^	*M_n_* PVDF (g/mol) ^a^	Đ ^b^
PM_23_-*b*-PVDF_77_	Linear diblock copolymer	28.6 K	6.6 K	22.0 K	PM: 1.12 PVDF: 1.29
PM_38_-*b*-PVDF_62_	Linear diblock copolymer	17.6 K	6.6 K	11.0 K	PM: 1.12 PVDF: 1.25
PM_23_PVDF_77_	Blend	-	5.6 K	7.6 K	
PM_38_PVDF_62_	Blend	-	5.6 K	7.6 K	
PVDF	Linear homopolymer	7.6 K	-	7.6 K	1.50 ^c^
PM-OH	Linear homopolymer	5.6 K	5.6 K	-	1.12 ^d^

^a^ All *M_n_* were determined by ^1^H NMR, toluene-d_8_, and DMF-d_7_ mixture; ^b^ Direct GPC characterization of PM-*b*-PVDF copolymers was impossible due to the difficulty in finding a common solvent for both blocks. The results given in the Table correspond to each block after hydrolysis of the junction point; ^c^ HT-GPC (trichlorobenzene as eluent, 145 °C, PS standards) for PM-OH and ^d^ GPC (dimethylformamide as eluent, 35 °C, PS standards).

**Table 2:** Melting and crystallization temperatures and enthalpies for each block copolymer, blend, and homopolymer sample studied.

Sample	Polymer	Rate (°C/min)	*T_m_*_,PM_ (°C)	*T_m,α_* (°C)	*T_m,β_* (°C)	*T_m,γ_* (°C)	*T_c_* (°C)	Δ*H_m_* (J/g)	Δ*H_c_* (J/g)
Homopolymer	PVDF	1	-	-	170.9	178.1	150.6	52.6	69.8
		5	-	173.5	168.2	-	144.0	53.8	60.4
		20	-	173.0	166.3	-	138.2	54.3	57.0
		60	-	172.5	165.0	-	129.3	53.8	58.5
PM_23_-*b*-PVDF_77_	PM	1	113.0	-	-	-	107.9	19.9	4.6
		5	112.2	-	-	-	105.6	25.1	3.4
		20	112.1	-	-	-	102.3	23.7	3.0
		60	111.9	-	-	-	98.3	24.3	1.6
	PVDF	1	-	170.9	-	-	147.8	67.1	67.0
		5	-	167.6	161.1	-	141.7	66.6	69.5
		20	-	166.1	158.9	-	135.9	70.6	71.6
		60	-	165.4	157.3	-	128.9	71.0	60.8
PM_38_-*b*-PVDF_62_	PM	1	114.4	-	-	-	108.4	38.4	25.6
		5	113.7	-	-	-	106.3	40.6	19.6
		20	113.4	-	-	-	103.4	43.2	18.8
		60	112.7	-	-	-	98.9	43.6	12.6
	PVDF	1	-	164.3	158.7	-	141.9	60.7	66.9
		5	-	162.1	155.2	-	137.6	57.4	72.3
		20	-	160.4	153.0	-	132.3	64.8	76.1
		60	-	159.1	150.6	-	124.2	70.4	65.7
PM_23_PVDF_77_	PM	1	100.5	-	-	-	92.7	24.6	37.5
		5	98.5	-	-	-	90.1	23.9	12.2
		20	97.8	-	-	-	86.1	13.1	10.9
		60	97.1	-	-	-	80.8	13.8	12.9
	PVDF	1	-	-	172.7	179.3	157.4	30.5	33.6
		5	-	174.4	170.7	176.9	152.5	37.3	38.9
		20	-	174.2	170.1	-	147.0	33.5	37.4
		60	-	173.4	167.8	-	141.0	35.0	37.7
PM_38_PVDF_62_	PM	1	100.9	-	-	-	94.3	17.2	17.9
		5	99.6	-	-	-	91.5	12.5	13.2
		20	98.1	-	-	-	86.8	20.5	14.1
		60	97.4	-	-	-	80.8	21.9	14.4
	PVDF	1	-	-	172.2	179.1	157.5	25.8	26.8
		5	-	174.5	170.2	176.7	151.5	25.8	29.5
		20	-	173.6	168.4	-	146.4	26.8	28.9
		60	-	173.1	167.3	-	139.8	27.1	29.7

**Table 3:** Values and description of the main FTIR bands for α, β, γ-phases for PVDF and PM.

Wavenumber (cm^−1^)	Phase	Description [61,62]
720	PM	C-C rocking deformation
796	α-PVDF	CH_2_ rocking
811	γ-PVDF	-
840	β-PVDF	CH_2_,CF_2_ asymmetric stretching vibration
976	α-PVDF	CH out of plane deformation
1214	α-PVDF	CF stretching
1232	γ-PVDF	CF out of plane deformation
1275	β-PVDF	CF out of plane deformation
1377	PM	CH_3_ symmetric deformation

**Table 4:** Arrhenius fit results for PVDF and its copolymers with PM.

Sample	$\tau_{0}$ (s)	$E_{A}$ (kJ/mol)	$\tau_{0}$ (s)	D	$T_{VFT}$ (°C)	$T_{g-BDS}$ (°C)
PVDF	2 × 10^−14±1^	42 ± 1	10^−14^	21 ± 1	−151 ± 1	−80 ± 1
PM_23_-*b*-PVDF_77_	3 × 10^−16±1^	48 ± 1			−154 ± 1	−85 ± 1
PM_38_-*b*-PVDF_62_	2 × 10^−16±1^	47 ± 1			−155 ± 1	−86 ± 1

## Data Availability

The data presented in this study are available upon request from the corresponding author.

## Supplementary Materials

The following are available online at https://www.mdpi.com/article/10.3390/polym13152442/s1, Figure S1: ^1^H NMR (500 MHz) spectrum of *CTA* in (CD_3_)_2_CO at 40 °C. Figure S2: ^1^H NMR (500 MHz) spectrum of Linear PVDF in DMF-d_7_ at 25 °C. Figure S3: ^19^F NMR (500 Hz) spectrum of Linear PVDF in DMF-d_7_ at 25 °C. Figure S4: GPC trace (DMF, 40 °C, PS standard) of linear PVDF (negative refractive index increment). Figure S5: Values of the intensity of the colours against the temperature during the cooling process at 20 °C/min using the Image J software of (a) PM_23_-*b*-PVDF_77_ and (b) PM_38_-*b*-PVDF_62_. Figure S6: Melting temperatures against crystallization temperatures with their respective linear fit to calculate the equilibrium melting temperature using the Hoffman–Weeks method. Table S1: Polymerization conditions and molecular characteristics of the linear PVDF synthesised by RAFT polymerization. Table S2: Equilibrium melting temperature (*T_m_*^0^) for the PVDF homopolymer, PVDF blends and PVDF block copolymers. Scheme S1: Synthesis of PM-b-PVDF Diblock Copolymer by Polyhomologation and ITP.
