# Supplementary material for: Phase Transitions in Poly(vinylidene fluoride)/Polymethylene-Based Diblock Copolymers and Blends
Source: Polymers (Basel). 2021 Jul 24;13(15):2442. doi: 10.3390/polym13152442 (PMC8348057; doi:10.3390/polym13152442)
Supplement: Supplementary file 1 [file polymers-13-02442-s001.zip › polymers-1309582-supplementary.pdf]

Phase transitions in Poly(vinylidene fluoride)/Polymethylene-Based Diblock Copolymers and Blends

Nicolás María [1], Jon Maiz [1,2,3,*], Daniel E. Martínez-Tong [2,4], Angel Alegria [2,4], Fatimah Algarni [5], George Zapzas [5], Nikos Hadjichristidis [5,*] and Alejandro J. Müller [1,3,4,*]

[1] POLYMAT, University of the Basque Country UPV/EHU, Avenida de Tolosa 72, 20018 Donostia-San Sebastián, Spain; nicolas.maria@polymat.eu

[2] Centro de Física de Materiales (CFM) (CSIC-UPV/EHU)-Materials Physics Center (MPC), Paseo Manuel de Lardizabal 5, 20018 Donostia-San Sebastián, Spain; danielenrique.martinezt@ehu.eus (D.E.M.-T.); angel.alegria@ehu.eus (A.A.)

[3] IKERBASQUE—Basque Foundation for Science, Plaza Euskadi 5, 48009 Bilbao, Spain

[4] Department of Polymers and Advanced Materials: Physics, Chemistry and Technology, University of the Basque Country UPV/EHU, Paseo Manuel de Lardizabal 3, 20018 Donostia-San Sebastián, Spain

[5] Polymer Synthesis Laboratory, KAUST Catalysis Center, Physical Sciences and Engineering Division, King Abdullah University of Science and Technology (KAUST), Thuwal 23955-6900, Saudi Arabia; fatimah.algarni@kaust.edu.sa (F.A.); georgios.zapsas@kaust.edu.sa (G.Z.)

* Correspondence: jon.maizs@ehu.eus (J.M.); nikolaos.hadjichristidis@kaust.edu.sa (N.H.); alejandrojesus.muller@ehu.es (A.J.M.)

Synthesis of linear polyvinylidene fluoride (PVDF) homopolymer

The synthesis of linear PVDF homopolymer has been accomplished by reversible addition–fragmentation chain-transfer (RAFT) polymerization of vinylidene fluoride (VDF) using S-benzyl O-ethylxanthate as chain transfer agent (CTA) and 1,1-bis(tert-butylperoxy)cyclohexane (Luperox® 331P80) as initiator in dimethyl carbonate at 80 ^{0}C.

Synthesis of RAFT Chain Transfer Agents

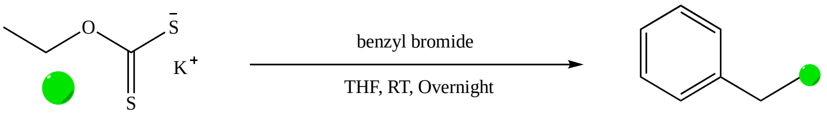

S-benzyl O-ethylxanthate (*CTA*). In a 100 ml round bottom flask, benzyl bromide (1 g, 5.84 mmol) and potassium ethyl xanthate (1.12 g, 7 mmol) were dissolved in anhydrous THF (50 mL). After degassing, the reaction mixture was stirred overnight at room temperature under argon. The reaction mixture was quenched with brine (100 mL) and extracted 3 times with dichloromethane (3x150 mL). The combined organic extracts were dried over $MgSO_4$, filtered, and concentrated by rotary evaporation. The *(CTA)* was obtained as a yellowish oil in >99% yield (1.2 g, 5.74 mmol). ^{1}H NMR (500 MHz, $(CD_3)_2CO$, δ (ppm), **Figure 1**): 7.26-7.42 (m, 5H, C_6**H_5**-), 4.64-4.68 (q, 2H, O-**CH_2**-CH_3), 4.42 (s, 2H, C_6H_5-**CH_2**-S), 1.39-1.42 (t, 3H, O-CH_2-**CH_3**). ^{13}C NMR (500 MHz, $(CD_3)_2CO$, δ (ppm): 213.66, 137.25, 130.10, 129.58, 128.49, 71.13, 40.17, 14.24.

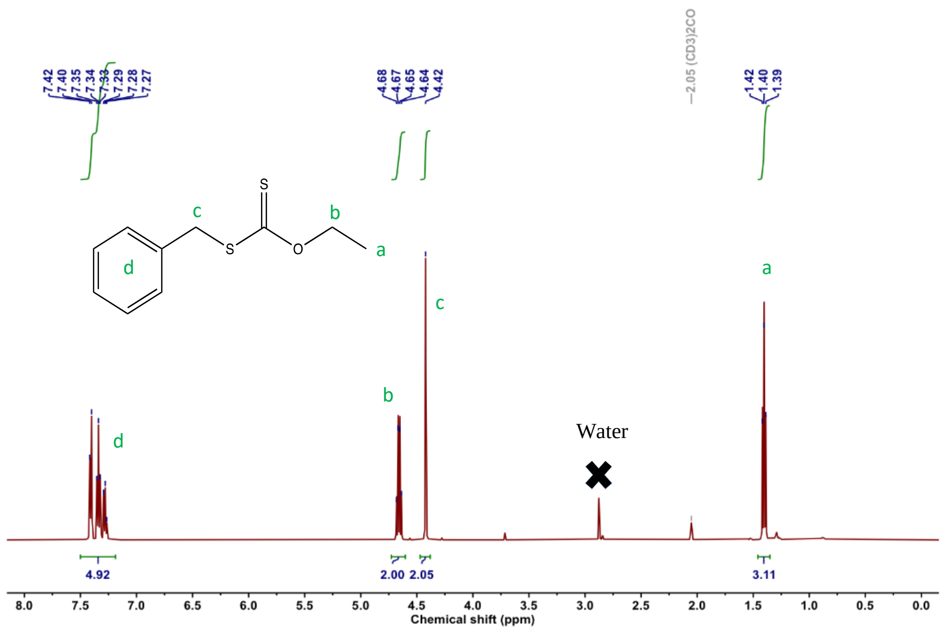

Figure S1. ^{1}H NMR (500 MHz) spectrum of ***CTA*** in $(CD_3)_2CO$ at 40 ^{0}C.

Synthesis of Linear PVDF

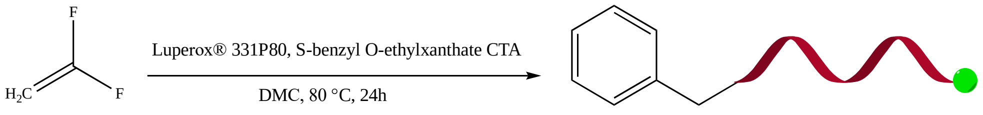

A Parr autoclave was filled with S-benzyl O-ethylxanthate, CTA (71.9 mg, 0.339 mmol) and 1,1-bis(tert-butylperoxy)cyclohexane (Luperox® 331P80). initiator (88.14 mg, 0.339 mmol) dissolved in dimethyl carbonate (75 mL). The reactor was cooled in a liquid nitrogen bath to condense VDF gas (21.7 g, 339 mmol) into an autoclave under weight control. It was then heated gradually up to 80 °C, and the evolutions of pressure and temperature were recorded. The reaction was stopped after 24 h, and the autoclave was cooled to room temperature and then placed in an ice bath. After the nonreacted monomer was purged, the reactor was opened and a colorless liquid was obtained. The solution was concentrated by rotary evaporation, precipitated in methanol, dried under vacuum for 24 h at 40 °C (7.4 g as a white powder). ^{1}H NMR (500 MHz, DMF-d_7, δ (ppm), **Figure 2**): 1.43-1.48 (t, CTA, –CF_2–CH_2–S(C=S)OCH_2–**CH_3**; –CH_2–CF_2–S(C=S)OCH_2–**CH_3**), 2.37 (t, PVDF, –CF_2–**CH_2–CH_2**– CF_2–,HH addition), 2.50 (t, CTA,C_6H_5-**CH_2**-), 3.03 (t, PVDF, –CF_2–**CH_2**– CF_2–,HT addition), 4.2 (t, PVDF– CF_2–**CH_2**–S–), 4.73-4.79 (q, CTA, –CF_2–CH_2–S(C=S)O**CH_2**–CH_3; –CH_2–CF_2–S(C=S)O**CH_2**–CH_3), 6.43 (tt, PVDF, –CH_2–CF_2–**H**), 7.23-7.39 (m, CTA, **–C_6H_5**).

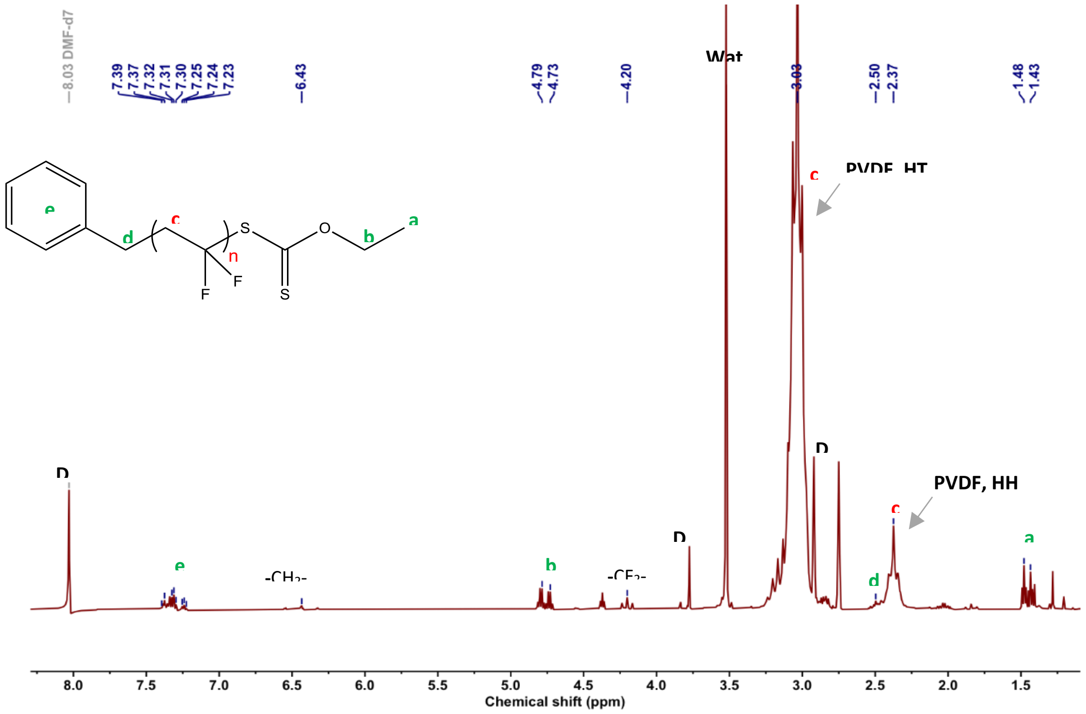

Figure S2: ^{1}H NMR (500 MHz) spectrum of Linear PVDF in DMF-d_7 at 25 ^{0}C

^{19}F NMR (500 MHz, DMF-d$_7$, δ (ppm), **Figure 3**): −116.97 (–CH$_2$–**CF$_2$–CF$_2$**–CH$_2$–, HH reverse addition), −115.72 (–CH$_2$–**CF$_2$**–H), −114.68 (–CH$_2$–**CF$_2$–CF$_2$**–CH$_2$–,HH reverse addition), −114.43 ((–CH$_2$–**CF$_2$–CF$_2$**–CH$_2$–S–), −114.04 (–CH$_2$–**CF$_2$–CF$_2$**–CH$_2$–S–), −108.74 (–**CF$_2$**–CH$_3$), −96.15 (–CH$_2$–CH$_2$–**CF$_2$**–CH$_2$–, TT reverse addition), −94.29 (CH$_3$–O–(C=O)–O–CH$_2$–CH$_2$–**CF$_2$**–), -93.46 (–CH$_2$–**CF$_2$**–CH$_2$–CF$_2$H), −92.79 (–CH$_2$–CH$_2$–CF$_2$–CH$_2$–**CF$_2$**–CH$_2$–CF$_2$–, regular HT addition), −92.35 (–CH$_2$–**CF$_2$**–CH$_2$–, regular HT addition).

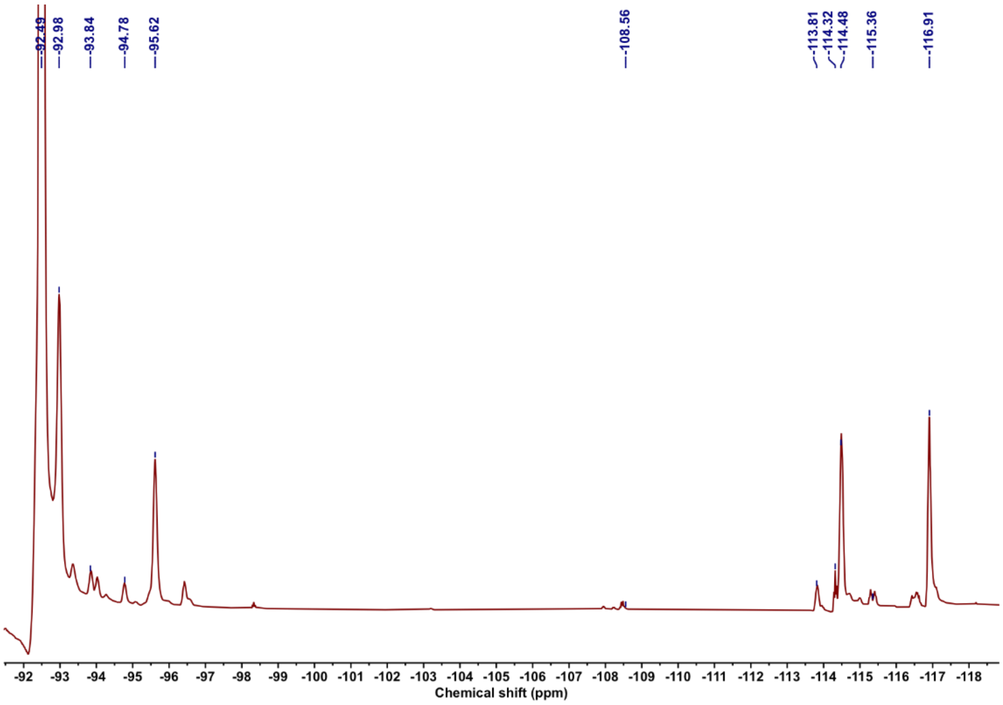

Figure S3: ^{19}F NMR (500 MHz) spectrum of Linear PVDF in DMF-d$_7$ at 25 ^{0}C

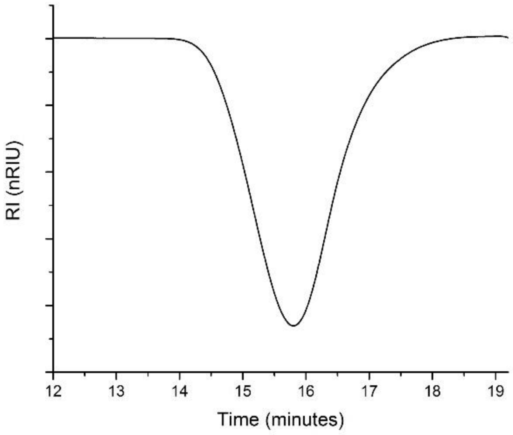

Figure S4: GPC trace (DMF, 40 ^{0}C, PS standard) of linear PVDF (negative refractive index increment)

The polymerization conditions and molecular characteristics of the synthesized PVDF are given in Table S1.

Table S1. Polymerization conditions and molecular characteristics of the linear PVDF synthesized by RAFT polymerization.

Polymer	Initiator	CTA	M	$[I]_0$:$[CTA]_0$:$[M]_0$	DP[a]	Mn[a] (g mol^{-1})	Đ[b]
Linear PVDF-	Luperox® 331P80	S-benzyl O-ethylxanthate	VDF	1: 1: 1000	120	7600	1.5

[a]Degree of polymerization and M_n were determined by ^{1}H NMR. [b]Determined by GPC in DMF, calibrated with linear PS standards.

The software Image J was used in order to quantify the change in the birefringence during the cooling at 20 ºC/min for both copolymers. The intensity was measured taking one picture each 10 ºC during the whole cooling process always measuring the same zone in all the pictures. Moreover, the intensity was normalized to obtain a values between 0 and 1. Figure S5 shows the intensity of the chosen zone against the temperature for both block copolymers. In both samples in the molten state the intensity is the lowest, when the temperature reaches 130-140 ºC the crystallization of the PVDF block starts and the intensity increases its value. When the cooling process continue and the value of the temperature is around 100 ºC there is a new change in the value of intensity that corresponds to the crystallization of the PM block. Therefore, the crystallization of the PM block inside the PVDF block spherulites already crystallized is demonstrated.

Synthesis of block copolymers

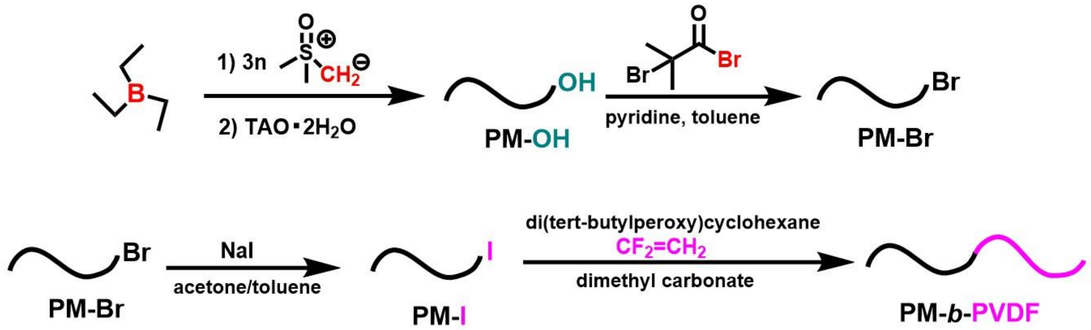

Scheme S1. Synthesis of PM-b-PVDF Diblock Copolymer by Polyhomologation and ITP.

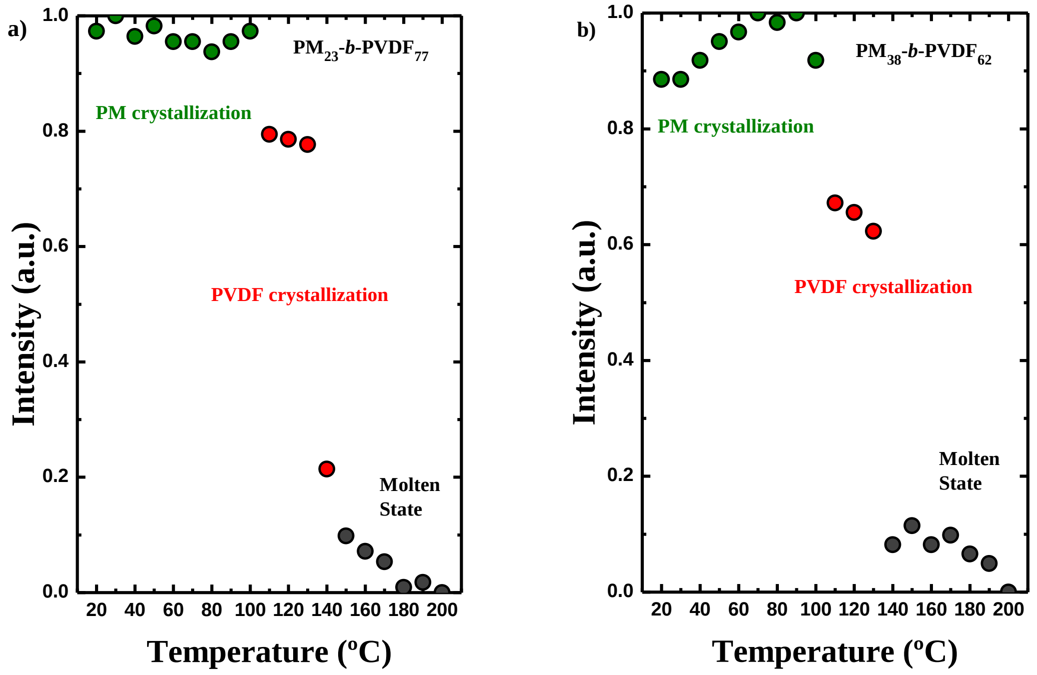

Figure S5. Values of the intensity of the colours against the temperature during the cooling process at 20 ºC/min using the Image J software of a) PM_{23}-*b*-$PVDF_{77}$ and b) PM_{38}-*b*-$PVDF_{62}$.

Using the isothermal crystallization temperature and the melting temperature obtained after the isothermal process, as Hoffman-Weeks theory predicted, it is possible to obtain the PVDF equilibrium melting temperature (T_m^0) for each sample. This method consists in using the melting temperatures obtained in the heating process after the isothermal crystallization procedure and plotting them against their respective crystallization temperatures obtaining a linear plot. When extrapolating this linear fit, the intersection with the T_m=T_c line is the equilibrium melting temperature [1,2]. The values for the T_m^0 of all samples containing PVDF are shown in Table S2. In the case of the diblock the melting peaks used to obtain the T_m^0 are from the melting of the α-phase, because is the only one stable during all the temperatures. However, for the homopolymer and the blends the α-phase does not exist so the melting peaks used to obtain the T_m^0 are from the β-phase in these cases. These values were used to plot Figures 9b and 10c in the main article. In our work the T_m^0 values are in a big range of 176-201 ºC according to the PVDF T_m^0 values reported in other works that are in an interval between 172-201 ºC [3,4]. In the Figure S6 are plotted the melting temperatures against their isothermal crystallization temperatures to calculate the equilibrium melting temperature with the cross with the diagonal line fitted.

Table S2. Equilibrium melting temperature *(T_m^0)* for the PVDF homopolymer, PVDF blends and PVDF block copolymers.

**Sample**	PVDF homopolymer	PM_{23}-*b*-$PVDF_{77}$	PM_{38}-*b*-$PVDF_{62}$	$PM_{23}PVDF_{77}$	$PM_{38}PVDF_{62}$
T_m^0 **(°C)**	176.8	176.4	177.1	199.5	200.6

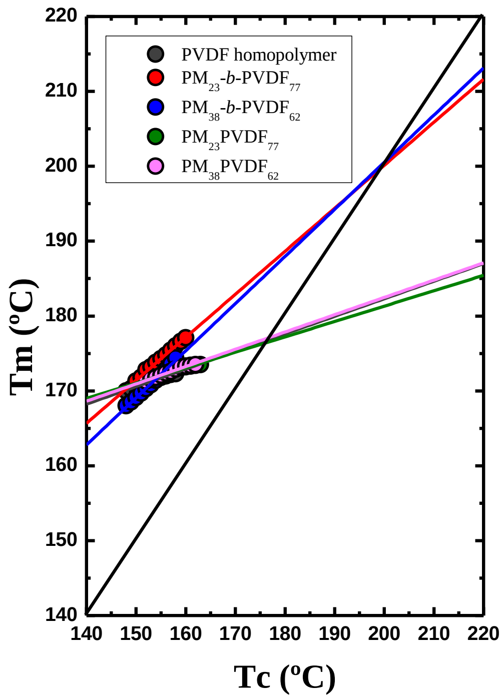

Figure S6. Melting temperatures against crystallization temperatures with their respective linear fit to calculate the equilibrium melting temperature using the Hoffman-Weeks method.

References

1. Hoffman, J.D.; Weeks, J.J. Melting process and the equilibrium melting temperature of polychlorotrifluoroethylene. *J. Res. Natl. Bur. Stand., Sect. A* **1962**, *66*, 13-28.
2. Marand, H.; Xu, J.; Srinivas, S. Determination of the Equilibrium Melting Temperature of Polymer Crystals: Linear and Nonlinear Hoffman−Weeks Extrapolations. *Macromolecules* **1998**, *31*, 8219-8229, doi:10.1021/ma980747y.
3. Kalivianakis, P.; Jungnickel, B.J. Crystallization-induced composition inhomogeneities in PVDF/PMMA blends. *Journal of Polymer Science Part B: Polymer Physics* **1998**, *36*, 2923-2930,doi:https://doi.org/10.1002/(SICI)1099-0488(19981130)36:16<2923::AID-POLB7>3.0.CO;2-R.

4. Xiao, Q.; Wang, X.; Li, W.; Li, Z.; Zhang, T.; Zhang, H. Macroporous polymer electrolytes based on PVDF/PEO-b-PMMA block copolymer blends for rechargeable lithium ion battery. *Journal of Membrane Science* **2009**, *334*, 117-122, doi:https://doi.org/10.1016/j.memsci.2009.02.018.
